# Does a novel bridging collar in endoprosthetic replacement optimise the mechanical environment for osseointegration? A finite element study

**DOI:** 10.3389/fbioe.2023.1120430

**Published:** 2023-06-05

**Authors:** Giulia Fraterrigo, Enrico Schileo, David Simpson, Jonathan Stevenson, Ben Kendrick, Fulvia Taddei

**Affiliations:** ^1^ IRCCS Istituto Ortopedico Rizzoli, Laboratorio di Bioingegneria Computazionale, Bologna, Italy; ^2^ Adler Ortho S.p.A., Cormano, Italy; ^3^ Royal Orthopaedic Hospital NHS Foundation Trust, Birmingham, United Kingdom; ^4^ Aston University Medical School, Aston University, Birmingham, United Kingdom; ^5^ Nuffield Orthopaedic Centre, Oxford University Hospitals Trust, Oxford, United Kingdom; ^6^ Nuffield Department of Orthopaedics, Rheumatology and Musculo-skeletal Science, University of Oxford, Oxford, England

**Keywords:** limb salvage surgery, endoprosthetic replacements, osseointegration, femur, collar, bone resorption, finite elements, contact modelling

## Abstract

**Introduction:** Limb-salvage surgery using endoprosthetic replacements (EPRs) is frequently used to reconstruct segmental bone defects, but the reconstruction longevity is still a major concern. In EPRs, the stem-collar junction is the most critical region for bone resorption. We hypothesised that an in-lay collar would be more likely to promote bone ongrowth in Proximal Femur Reconstruction (PFR), and we tested this hypothesis through validated Finite Element (FE) analyses simulating the maximum load during walking.

**Methods:** We simulated three different femur reconstruction lengths (proximal, mid-diaphyseal, and distal). For each reconstruction length one in-lay and one traditional on-lay collar model was built and compared. All reconstructions were virtually implanted in a population-average femur. Personalised Finite Element models were built from Computed Tomography for the intact case and for all reconstruction cases, including contact interfaces where appropriate. We compared the mechanical environment in the in-lay and on-lay collar configurations, through metrics of reconstruction safety, osseointegration potential, and risk of long-term bone resorption due to stress-shielding.

**Results:** In all models, differences with respect to intact conditions were localized at the inner bone-implant interface, being more marked in the collar-bone interface. In proximal and mid-diaphyseal reconstructions, the in-lay configuration doubled the area in contact at the bone-collar interface with respect to the on-lay configuration, showed less critical values and trends of contact micromotions, and consistently showed higher (roughly double) volume percentages of predicted bone apposition and reduced (up to one-third) percentages of predicted bone resorption. In the most distal reconstruction, results for the in-lay and on-lay configurations were generally similar and showed overall less favourable maps of the bone remodelling tendency.

**Discussion:** In summary, the models corroborate the hypothesis that an in-lay collar, by realising a more uniform load transfer into the bone with a more physiological pattern, creates an advantageous mechanical environment at the bone-collar interface, compared to an on-lay design. Therefore, it could significantly increase the survivorship of endo-prosthetic replacements.

## 1 Introduction

Limb-salvage surgery using endoprosthetic replacements (EPRs) are frequently used to reconstruct segmental bone defects after *en-bloc* excision of malignant bone tumours of the proximal and distal femur and are also increasingly used for failed osteosynthesis and arthroplasty with significant bone loss (Chao et al., 2004; Myers et al., 2007; Jeys et al., 2008; Dean et al., 2012). Longevity of the reconstruction is, however, a major concern, especially in young and active patients who place high demands on their prostheses (Jeys et al., 2008; Farfalli et al., 2009).

In bone sarcoma, advances in chemotherapy have led to prolonged life expectancy meaning that reconstructions must be long-lasting if they are to avoid multiple revision operations (Grimer et al., 2016). EPRs permit early weight-bearing and are associated with shorter operative times and without the disease transmission risk associated with allograft reconstructions also indicated for segmental bone loss, thus EPRs are an attractive option for elderly, co-morbid patients (Khajuria et al., 2018).

Complications are common after EPRs due to multiple patient and surgery factors not limited to long surgical procedures, large soft-tissue resections, neo-adjuvant chemo-radiotherapy. Although these implants are widely used the rate of complications for any reason remains five to ten times higher than rates seen following routine total joint arthroplasties (Parvizi et al., 2007; Coathup et al., 2015). In any case, as classified by Henderson et al. (2014) complications across revision and sarcoma surgery with EPRs, both mechanical (soft tissue failure, aseptic loosening, structural failure) and non-mechanical (local recurrence, infections) are common (Jeys et al., 2008; Farfalli et al., 2009; Shehadeh et al., 2010). Aseptic loosening is a common mode of failure, accounting for 25% of revisions in a series of 661 EPRs for oncological indications (Jeys et al., 2008). Other studies have highlighted the rate of aseptic loosening ranges from 2.9% to 28.6% after four to 10 years (Unwin et al., 1996; Torbert et al., 2005).

Loosening of EPRs is associated with loss of cortical bone, initially at the point of contact between the bone and the collar of the prosthesis, then progressing along the stem (Mumith et al., 2017). Having a region of ingrowth on the prosthesis next to the bone at the site of transection promotes extracortical bone bridging (ECBB) (Chao et al., 2004; Mumith et al., 2017). Bone from the cortex at this site grows out and over the collar of the prosthesis. Osseointegration at the collar of the EPR has the potential to reduce the risk of aseptic loosening by improving stress transfer between the implant and bone. It is also thought that ECBB reduces aseptic loosening by sealing the bone/prosthesis interface, which in turn can prevent wear debris and synovial fluid from gaining access to the interface, decreasing the risk of osteolysis (Ward et al., 1993; Mumith et al., 2017).

A more porous collar structure might permit greater ingrowth of bone directly from the transected cortex thereby improving osseointegration (Bram et al., 2006). Although block porous metal has been used successfully to treat metaphyseal defects around revision arthroplasty of the knee (Kamath et al., 2015) it has not been used to encourage ECBB with EPRs. In animal models, a porous collar allows the direct ingrowth of more bone and is superior to current designs which rely on surface ongrowth and ECBB (Mumith et al., 2017). Conventional collars rely on bone bridging externally to the collar because these collars sit on top of the bone at the resection site, described as ‘on-lay’ collars. A novel collar design utilising a porous on-lay collar with a porous endosteal ‘in-lay’ sleeve has the theoretical advantage of immediate primary stability from the press-fit of the endosteal sleeve and early osseointegration *via* endosteal cellular growth. The mechanical environment is hypothesised to be further optimised with an in-lay collar and thereby maximising ECBB at the implant-bone interface.

Finite element analysis (FEA) has demonstrated that the stem-collar junction is the region subjected to the highest stresses (Fromme et al., 2017) and clinical studies indicate this region to be the most common site of mechanical failure (Agarwal et al., 2010). In addition, the mechanical environment around the collar is a key factor on whether bone will grow onto the collar, or resorb, leading to aseptic loosening. A lack of osseointegration can lead to stress shielding whereby more of the stress is transferred from the collar of the implant into the tip of the stem. This can lead to bone resorption at the implant-collar interface and ultimately in stem fracture and complex revision surgery. ECBB where it integrates with the surface of the implant allows for a more physiological load transfer and reduced stress shielding (Fromme et al., 2017). Therefore, maximising the ECBB at the implant collar to reduce the incidence of aseptic loosening and increase EPR longevity would be beneficial to patients.

It is not practical to use experimental measurements to directly measure the strain distributions in the bone *in-vivo*, but experimental limitations can be overcome by using Finite Element (FE) analysis. FE models can be used to optimise implant design and verify the design intent of a new prosthesis before long-term clinical data has been recorded. Our study hypothesis was that an in-lay collar would demonstrate advantageous biomechanics around the collar and would be more likely to promote bone ongrowth. The analysis was carried out using a fully validated FE model simulating an instance during a high intensity functional activity.

## 2 Methods

### 2.1 Overview

**FIGURE 1:**
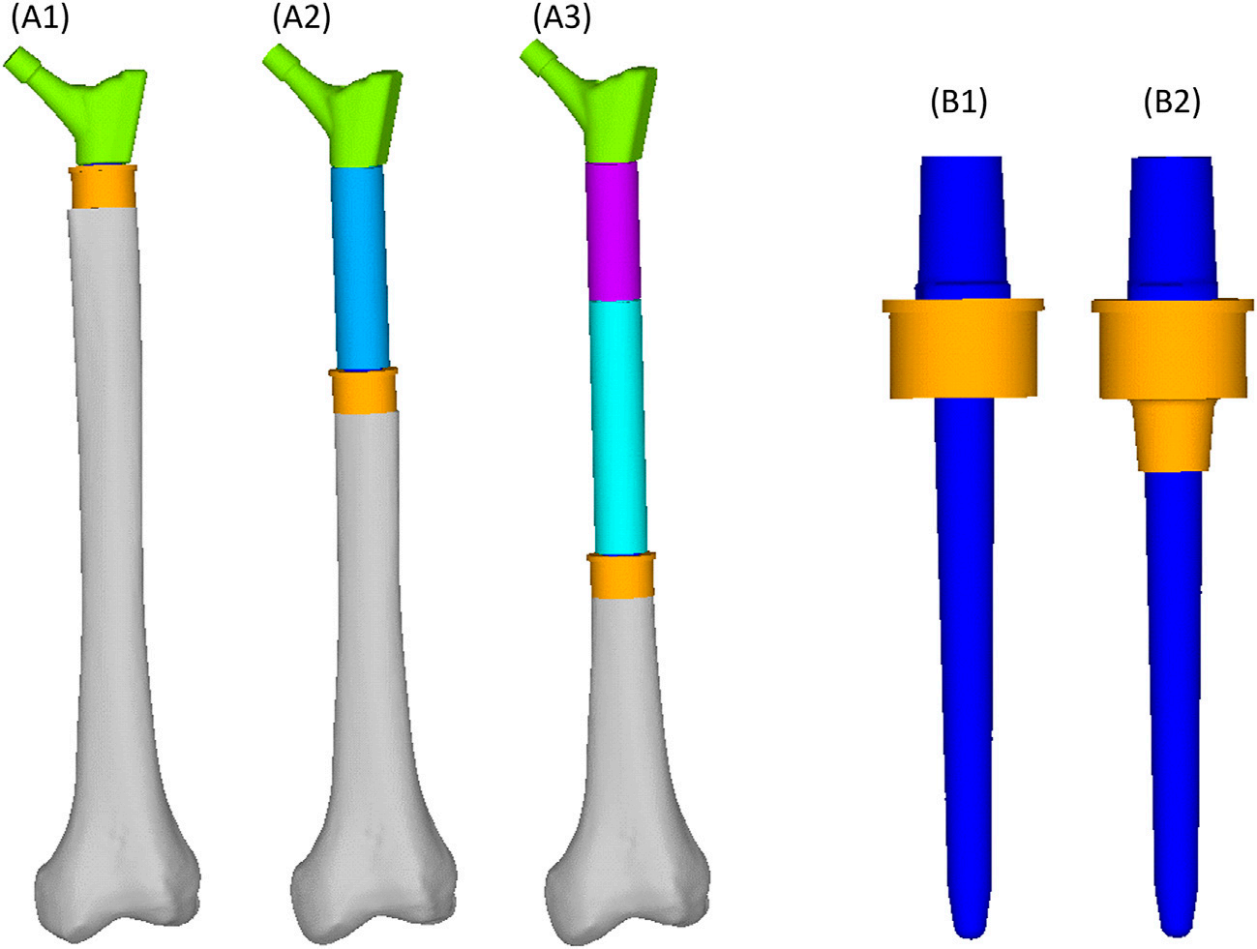
Geometry of the femoral reconstructions and concept of the two collar configurations investigated in this study. From left to right: Proximal PFR **(A1)**, Mid-diaphyseal PFR **(A2)**, Distal PFR **(A3)**; Prosthesis stem (blue) joined with on-lay **(B1)** and in-lay **(B2)** collar (orange).

We simulated three different reconstruction lengths (proximal, mid-diaphyseal, and distal) covering almost all possible PFR cases (Figure 1). For each reconstruction length one in-lay and one on-lay collar model was built and compared. All reconstructions were virtually implanted in a single femur, carefully chosen to represent the average of the population that may receive a PFR. Personalised FE models were built for the intact case and for all simulated PFR cases. In the implanted models we simulated immediate post-operative conditions. The FE models were derived from CT data and virtual implantation information, according to a validated modelling procedure (Schileo et al., 2008; Schileo et al., 2014) that included contact interfaces where applicable (Viceconti et al., 2000; Taddei et al., 2010). Results were analysed to compare the mechanical environment in the in-lay and on-lay collar configurations, deriving metrics of post-operative reconstruction safety/osseointegration potential, and of long-term bone resorption due to stress-shielding.

### 2.2 Average femur

**FIGURE 2:**
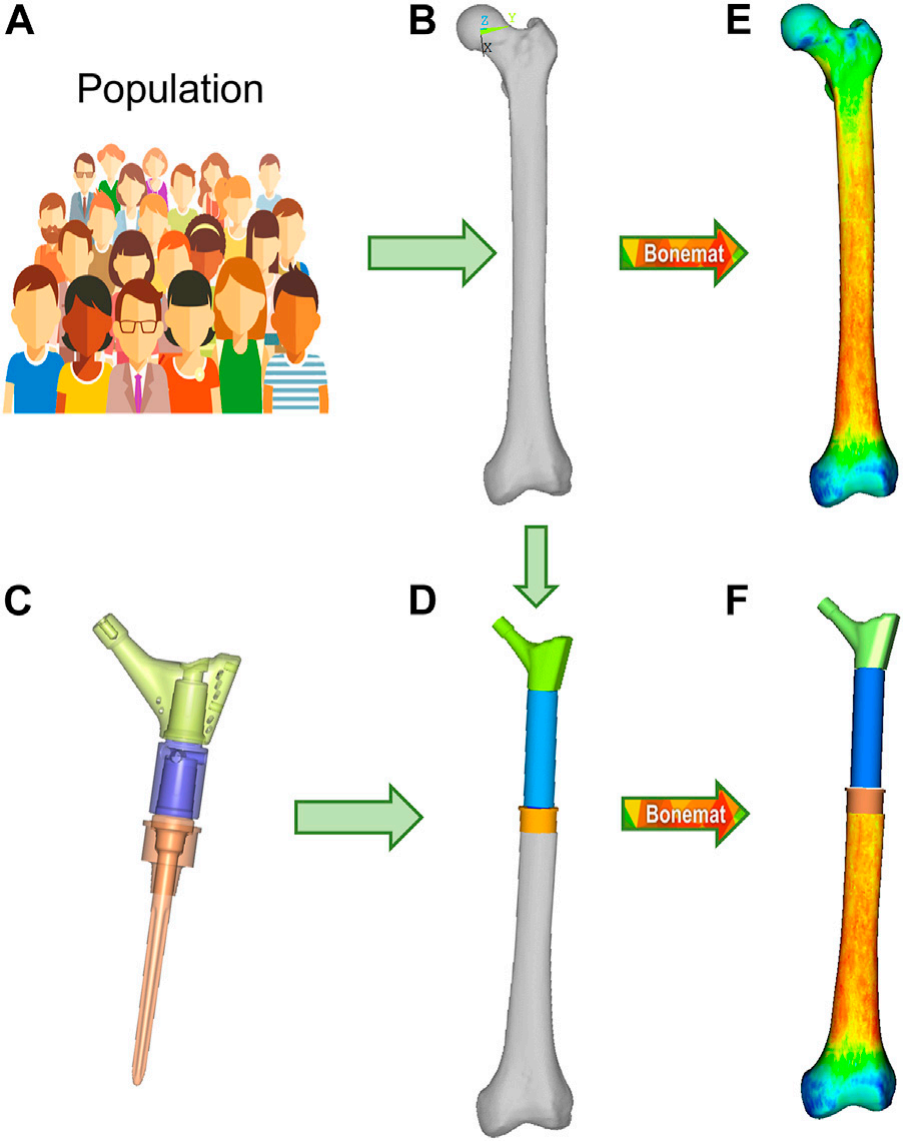
Finite element modelling workflow, described for the M-PFR configuration. From a group representative of anatomical and density variations in a population **(A)**, the mean femur is extracted **(B)**, and implanted with the PFR (**(C)**, with inner details on juctions, and **(D)**). Both intact and implanted geometries are meshed, and CT-based material properties are assigned **(E,F)**.

The average representative femur from this study was chosen from an already presented CT database (Taddei et al., 2014) of 200 femurs showing normal anatomy and absence of visible osteoarthritis signs (Grade 0 according to Altman and Gold (2007)). That database was already shown to be representative of the anatomical proximal femur variability in terms of diaphysis biomechanical length (from intertrochanteric to condylar saddles), femoral neck length, cervico-diaphyseal (CCD) angle, anteversion angle, compared to the widest available reports (Noble et al., 1995; Sugano et al., 1999; Toogood et al., 2009) (Table 1). Given that the PFR interface with bone happens in the diaphysis, we complemented these metrics adding two parameters measured on the diaphyseal bone cortex, i.e., cortical thickness and estimated periosteal radius. We measured these two additional parameters with an in-house software application developed from an open-source framework (ALBA, https://github.com/IOR-BIC/ALBA), averaging measurements from four radial profiles traced from the centroid of on a horizontal section at the femoral isthmus, excluding the linea aspera. Then, since clinical cases for PFR (oncologic or trauma) are prevalent at young age, we first restricted the database to young (18–40 years old) and densitometrically normal (CT-derived DXA T-score close to zero) cases, then chose the subject with the closest-to-average femoral geometry (Table 1; Figure 2B).

**TABLE 1:** Femur geometry: basic descriptive statistics of population distribution over 200 normal anatomies from (Taddei et al., 2014), and values of the femur used in this study.

	Biomech. Length (mm)	Neck Length (mm)	Anteversion Angle (°)	CCD angle (°)	Cortical Thickness (mm)	Diaphyseal radius (mm)
Mean (SD)	407 (29)	39.2 (4.7)	13.3 (8.4)	126.5 (7.3)	5.0 (0.8)	12.1 (1.2)
Min-Max	348–490	26.9–51.5	0.6–45.5	104.1–145.0	3.8–7.1	9.4–14.4
This study	409.5	38.7	7.6	121.8	5.8	12.8

### 2.3 Proximal femur reconstruction (PFR)

The Pantheon Proximal Femoral Reconstruction system (Adler Ortho S.p.A, Italy) was used in this study. Virtual implantation of all PFR cases (in-lay and on-lay collar configurations for proximal, mid-diaphyseal and distal reconstructions) was performed on CT data, with the aim to prioritise the best possible fit of prosthetic and intact femoral head centre and hence maximise the similarity of lever arms in the intact and implanted configurations (Figure 2D). A fit of the femoral head centre always below 4 mm could be achieved.

The PFRs in this study have a modular concept, therefore: the proximal reconstruction (P-PFR) was assembled with a femoral neck component, directly linked to the collar and stem (which are also two separate components, assembled through a taper junction); a shaft was added to achieve the mid-diaphyseal reconstruction (M-PFR); two shaft components were used to realize the distal reconstruction (D-PFR).

Cemented fixation of the prosthesis stem (not of the in-lay or of the on-lay collar) was simulated for all PFRs, through a uniform polymethylmethacrylate (PMMA) cement layer of 1 mm (equal to the nominal thickness indicated by the manufacturer for the implants) surrounding the stem from the collar end to just distal to the stem tip.

### 2.4 Finite element modelling

#### 2.4.1 PFR

The geometry files of the virtual implantation in NURBS format were defeatured to permit a smooth meshing phase, without omitting any mechanically relevant detail apart from the deletion of shallow recesses around the stem intended to host cement and thus act as anti-rotational features. These were accounted for in the contact model (Section 2.4.4 below).

A 10-node tetrahedral mesh was generated with an average edge length of 1.5 mm (Hypermesh v.21.0, Altair Engineering Inc., United States). To model the real assembly condition of the PFRs and avoid the simulation of innaturally bulky and stiff prostheses, nodes of the modular components (neck—shaft(s) where applicable—collar—stem) were tied at the actual sites where taper junctions occur (reflecting a condition of stable taper junctions). Titanium alloy material properties (Ti6Al4V, Young’s modulus 110 GPa, Poisson’s ratio 0.36) were assigned to PFR elements. The thin layer of trabecular titanium covering the surface of the on-lay collar was not assigned specific reduced properties because: i) its thinness hinders a real effect on the overall resistant section of the collar; ii) its osteoconductive and osteointegrating effects are likely due to its surface organisation rather than to the reduction of the elastic modulus towards a bone-like material, as trabecular titanium cells produced by the same manufacturer for custom made 3D reconstructions showed an effective elastic modulus of 97 GPa (i.e., not far from that of the bulk alloy) when mechanically tested (La Barbera et al., 2019).

#### 2.4.2 Femur

We developed six models of the femoral host bone to be coupled to P-PFR, M-PFR and D-PFR in the on-lay and in-lay collar configurations. To permit an element-by-element comparison of implanted and intact conditions, we built six corresponding intact models, isotopological to PFR models in the part where the PFR is inserted into the diaphysis.

Contours of the whole femur were identified through segmentation of the original CT data (Mimics 21, Materialise NV, BE) and then used to obtain a mathematical representation of the surfaces (Geomagic Studio v.7, Raindrop Geomagic Inc., United States). To generate the femur models, we resected the intact femur and subtracted the stem, collar, and cement components with a Boolean operation. A 10-node tetrahedral mesh was generated, setting an average element edge length of 1.5 mm on the surface (HyperMesh 21, Altair Engineering, Inc., United States). Subject-specific bone properties were assigned to bone elements: radiological density was obtained by CT calibration (European Spine Phantom, QRM Gmbh, DE), followed by radiological-to-ash density (linear relationship (Schileo et al., 2008) and ash-to-wet density transitions (fixed 0.6 ratio (Schileo et al., 2008)); wet apparent density was then transformed in Young’s modulus based on a density-elasticity relationship (Morgan et al., 2003). A homogeneous/constant Poisson ratio of 0.3 was set to all the mesh elements (Schileo et al., 2008). Material mapping from CT voxels to finite elements was performed with the Bonemat algorithm (freely available at www.bonemat.org), that provides an average Young’s modulus for each element of the mesh, performing a numerical integration of voxel properties extracted from the CT images (Taddei et al., 2007) (Figures 2E,F).

#### 2.4.3 Cement

**FIGURE 3:**
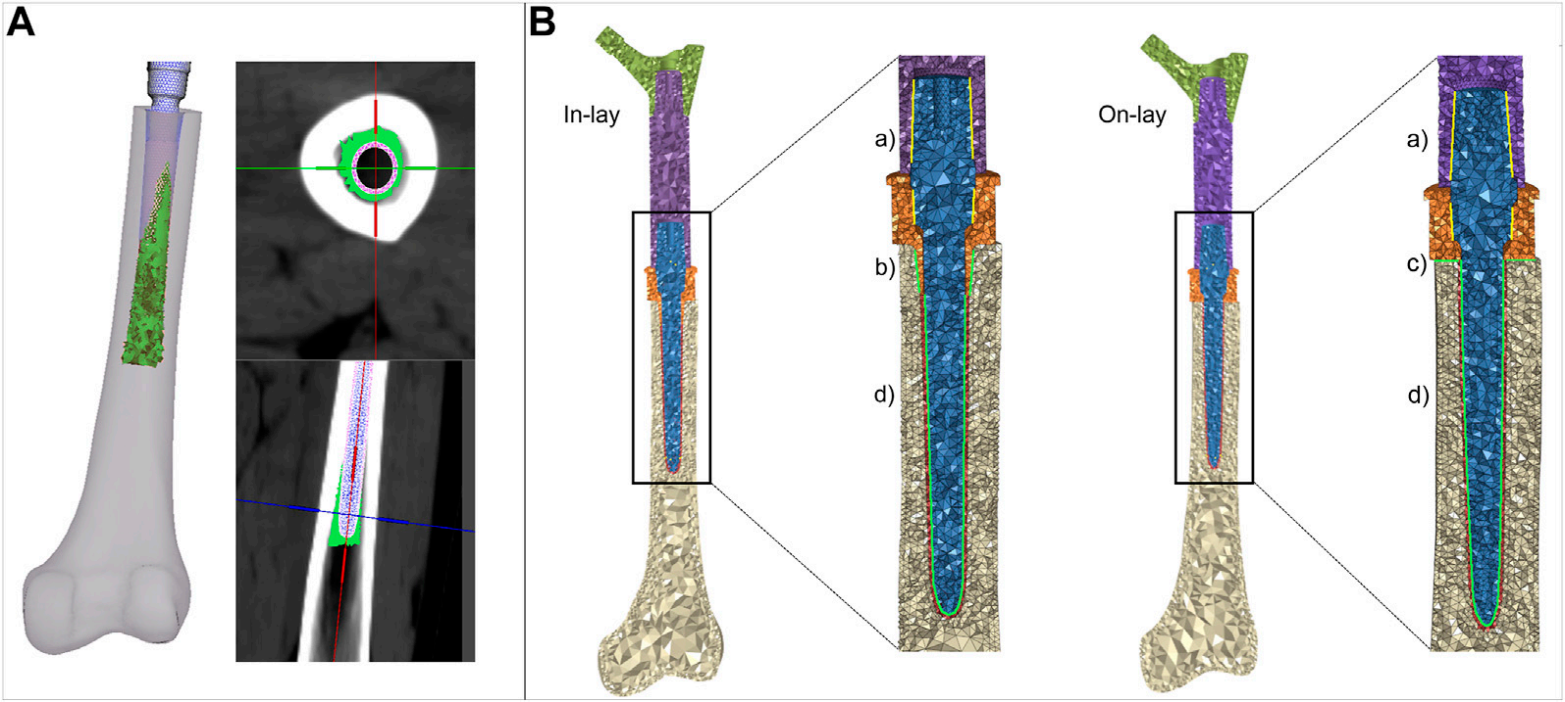
**(A)** details of the procedure to simulate cement interdigitation with porous bone. **(B)** details of contact interfaces for the in -lay and on-lay designs, representing bonded interfaces within the PFR **(Ba)**, and frictional interfaces between in-lay collar and bone **(Bb)**, on-lay collar and bone **(Bc)**, and stem and cement **(Bd)**.

The uniform 1 mm cement layer was meshed with 10-node tetrahedral elements. An exact match of cement/bone and cement/implant interfaces could be achieved proximally at the resection site, but as the stem extended inwards the uniform 1 mm cement layer was only sparsely in direct contact with the endosteum. To mimic the actual distribution of bone cement in the diaphysis canal, we allowed cement to i) circumferentially expand around the stem, interdigitating with bone elements of the endosteal surface that had a Young’s modulus lower than 2 GPa, ii) distally expand down to 5 mm from the stem tip (Figure 3A). Cement elements were assigned a Young’s modulus of 2.5 GPa and a Poisson ratio of 0.4.

#### 2.4.4 Interfaces

We intended to simulate a short term post-operative condition, where osseointegration is still to be achieved. Consequently: i) we modelled frictional contact interactions between collar and bone as a function of the surface characteristics of the collar material; ii) we modelled contact between stem and cement but considered bone and cement bonded. A schematic of contact interfaces is provided in Figure 3B.

Contact was numerically implemented in ANSYS (ANSYS APDL v.2020 R1, Ansys Inc., United States), where all models were solved. We adopted an augmented Lagrangian approach and used face-to-face contact elements with large sliding formulation (Viceconti et al., 2000). We built meshes ensuring isotopology of contact and target faces, and we forced a perfect initial match between contact surfaces neglecting any gap or penetration at the beginning of the loading step.

##### 2.4.4.1 Collar-bone

For the traditional on-lay collar design, which usually has a moderately rough surface finish we set a friction coefficient of 0.5 (as derived from Biemond et al. (2011) for sandblasted coatings) at the interface between the collar and the resected bone surface. For the new in-lay collar: i) we set the friction coefficient to 0.7 to simulate a metal trabecular surface (Biemond et al., 2011); ii) we simulated initial contact between the bone and the sleeve portion of the collar inserted into the diaphysis, leaving the collar ring facing the bone resection plane initially detached from it. This contact setting was intended to replicate the actual condition, which is most likely to occur, where an ideal fit of both the ring and sleeve portion of the collar is almost impossible to be obtained, and the collar fit would likely be privileged operationally.

##### 2.4.4.2 Stem-cement

Contact between the PFR stem and surrounding cement was assumed to have a friction coefficient of 0.3 (Nuño et al., 2006). To compensate for the defeaturing of the anti-rotational features we defined a local cylindric reference system and implemented an orthotropic coefficient of friction, increasing the circumferential coefficient to 0.9, and leaving the axial and transversal ones to 0.3.

#### 2.4.5 Model verification

The chosen average element size of 1.5 mm permitted an accurate discretisation of bone-implant interfaces and can be considered at convergence in the computation of: i) bone strains, as a mesh size of 3 mm showed convergence in a similar study (Helgason et al., 2008); ii) contact results, as a mesh size of 2 mm was used in a previous validation work in a contact model of implanted bone conditions (Taddei et al., 2010).

Mesh quality check in the bone (complex shape, heterogeneous) and cement components showed an aspect ratio over 3 and/or volumetric skew over 0.6 only in 1% of elements, with all angles between edges in the range 15°–125°. Mesh quality was optimal also at contact interfaces (only 2 elements with aspect ratio over 3, all angles between edges in the range 15°–125°). Mesh quality was slightly worse in the prosthesis, but deemed acceptable as the 1% of elements with aspect ratio over 5 or volumetric skew over 0.7 did not belong to interfaces as internal taper junctions or stem and were located in areas of little interest to our study.

At contact interfaces, residual penetrations at the end of the solution step were checked to be at least one order of magnitude lower than the observed contact sliding.

All material models were linear elastic. We did not include a post-elastic phase in material modelling as we were interested in the elastic response during regular exercise, and in verifying that no component would exceed the elastic limit. Also, validated models from which the modelling procedure was taken had used linear elastic materials.

### 2.5 Boundary conditions

#### 2.5.1 Constraints

**FIGURE 4:**
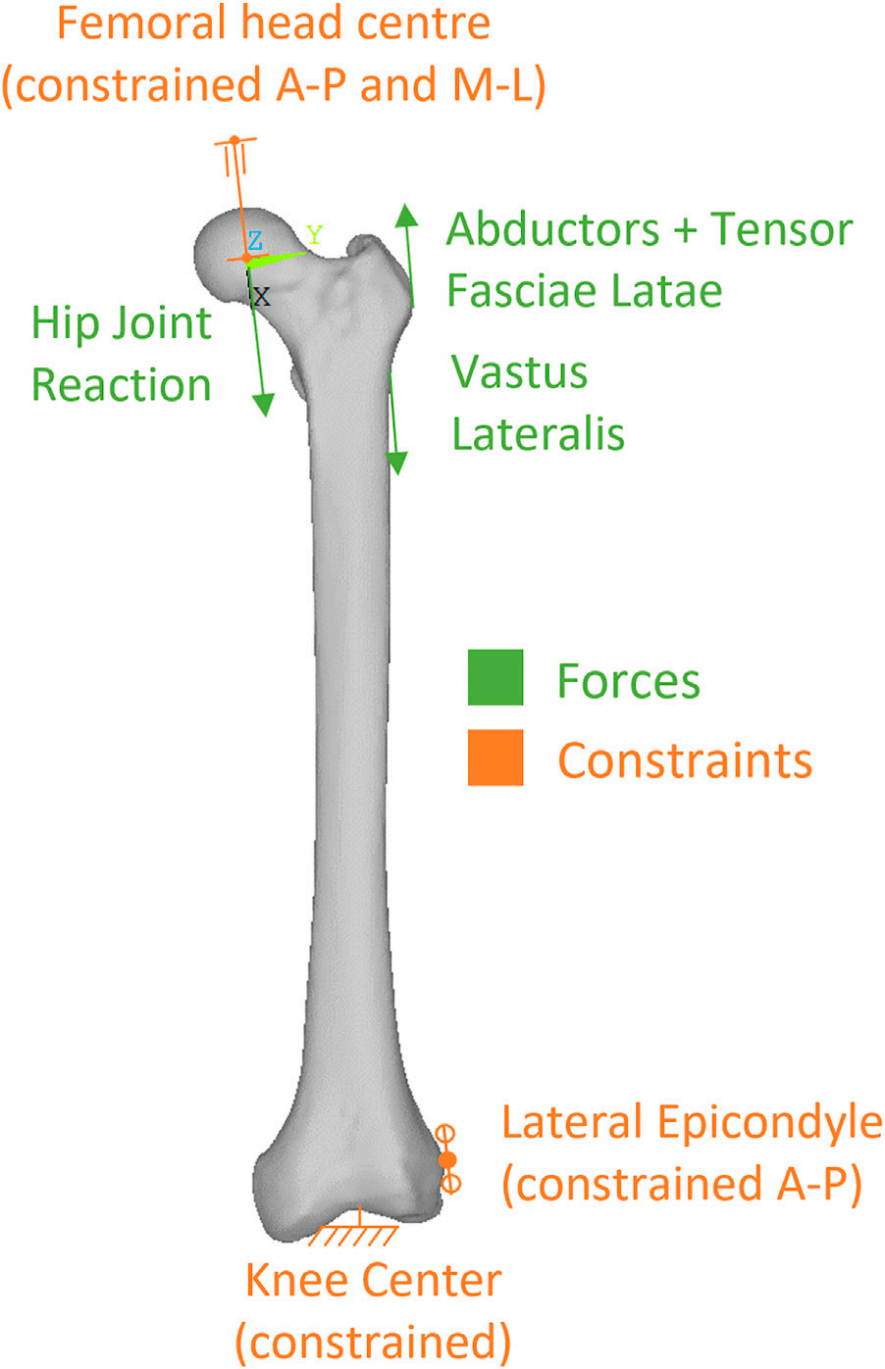
Description of the loads and constraints applied to simulate level walking.

Physiological-like constraints were applied to femur models according to the scheme proposed by (Speirs et al., 2007), that our research group has already applied to another femur reconstruction study (Valente et al., 2017). Knee stability in that scheme is obtained constraining in antero-posterior direction a single node at the lateral epicondyle, and fully constraining a single node in the knee centre (midpoint between epicondyles) (Figure 4). To permit femoral bending, the femoral head centre was constrained to move along the biomechanical axis of the femur that ideally joins the hip (femoral head) centre with the knee centre.

#### 2.5.2 Loads

We simulated loads on the bone from a standard level walking task, because walking is likely to be the most frequent motor task performed, thus the most suitable to investigate bone remodelling around the implant. An average estimate of physiological loads during walking was obtained from the work of Heller et al. (2005). We adopted the schematization of musculoskeletal loads proposed by Heller et al. (2005) because it is rather simple, yet validated against loads from instrumented prostheses, and already used in preclinical testing of joint prosthesis (Martelli et al., 2011a). In brief, a hip reaction force is applied (at the femoral head centre), equilibrated by muscle forces from abductor and tensor fascia latae (acting at the greater trochanter), and vastus lateralis (acting on the proximal femur diaphysis) (Figure 4). As muscles are usually successfully reattached to metal surfaces or adjacent tissues at the end of the surgical reconstruction trying to reproduce their original position, attachment locations were kept also in the PFR models. Force values in the original work were given in percentage of bodyweight and we thus scaled them to the weight (86 kg) of the modelled subject (Table 2).

**TABLE 2:** Applied loads.

Force (Application point)	Med-Lat (N | BW%)	Ant-Post (N | BW%)	Vertical (N | BW%)
Hip Joint Reaction (Femoral head centre)	456 N | 54%	−277 N | −32.8%	1934 N | 229.2%
Abductors + Tensor Fasciae Latae (Greater Trochanter)	−546 N | −64.7%	128 N | 15.2%	−681 N | −80.7%
Vastus Lateralis (Lateral lip of the linea aspera)	8 N | 0.9%	156 N | 18.5%	784 N | 92.9%

### 2.6 Analysis metrics

For each simulated PFR configuration we computed, according to the classification proposed by Martelli et al. (2011b): i) the percentage of interface area in contact under load and the interfacial sliding micromovements to quantify the risk of short-term aseptic loosening (risk thresholds set at 50 μm for implant-cement (Zhou et al., 1989) and 150 μm for implant-bone interfaces (Pilliar et al., 1986) respectively); ii) the longitudinal, principal and circumferential (hoop) bone strains around the implant to quantify the risk of bone failure (damage thresholds set at 0.73% for tensile and hoop strains, at 1.04% for compressive strains (Bayraktar et al., 2004); iii) the Von Mises equivalent stress on the whole PFR to verify its fatigue safety (fatigue limit set to 400 MPa as a conservative estimate from the data in Long and Rack, (1998)); iv) a strain-energy based indicator of possible positive (apposition) or negative (resorption) bone remodelling around the collar-bone interface. The indicator of bone remodelling tendency was defined (Martelli et al., 2011b) as:

$$R_{BRES}=\frac{\frac{\left(S-S_{ref}\right)}{S_{ref}}*100}{75}$$

where:

S = strain energy density per unit of mass.

S_ref_ = homeostatic reference (i.e., the S of intact femur) (Huiskes et al., 1992).

According to Kuiper and Huiskes (1997) values of R_BRES_ higher than 100 (i.e., strain energy densities over 175% of the intact reference) were considered indicative of bone apposition, values lower than −100 (i.e., strain energy densities lower than 25% of the intact reference) of bone resorption, and values between −100 and 100 (i.e., strain energy densities between 25% and 175% of the intact reference) of homeostasis.

An element-specific calculation was possible as for each PFR model we had built a corresponding isotopological intact model. This indicator was evaluated considering bone volumes of interest around the collar, as depicted in Table 4. A first VOI A) comprised the whole volume of interest around the sleeve portion of the in-lay collar (and corresponding volume in the on-lay configuration). This VOI was then eroded to consider B) the first two layers of bone elements extending radially from the implant, and then C) only the first layer of elements in contact with the implant.

For all PFR lengths, we compared the in-lay and on-lay collar configuration to each other, and each one to the intact femur condition when analysing bone strains and bone remodelling.

## 3 Results

### 3.1 Contact status

Contact status results are summarised in Table 3. In the P-PFR and M-PFR, the in-lay configuration doubled the area in contact at the bone-collar interface with respect to the on-lay configuration (32% vs. 16% in P-PFR, 38% vs. 19% in M-PFR). Collar contact areas always below 40% indicated that the lever exerted by the stiffer overlaying PFR did not permit to evenly distribute load all around the collar. Nonetheless, the larger area in contact in the in-lay collar translated in a more even distribution of load in the in-lay stem, so that larger in-lay than on-lay stem portions were in contact with cement. As a result, the total area in contact under load (summing the collar-bone and stem-cement interfaces) was >60% for the in-lay, and <40% for the on-lay configuration. Full contact maps for each PFR configuration can be found in Supplementary Appendix S1.

**TABLE 3:** Percentage of area in contact under load and maximum sliding micromotion at bone-implant and stem-cement interfaces.

		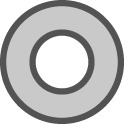 On-lay collar			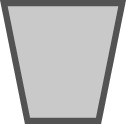 In-lay collar		
		Ring/Bone	Stem/Cement	Total	Sleeve/Bone	Stem/Cement	Total
P-PFR (Proximal)	% area in contact	16	38	36	32	73	64
	Max. sliding micromotion (µm)	46	77	—	76	37	—
M-PFR (Mid-diaphysis)	% area in contact	19	40	38	38	64	60
	Max. sliding micromotion (µm)	52	68	—	69	34	—
D-PFR (Distal)	% area in contact	34	31	31	16	37	33
	Max. sliding micromotion (µm)	26	26	—	54	33	—

Conversely, in D-PFR the on-lay collar showed larger contact area at the collar-bone interface, so that the overall area in contact was similar for both models. Notably, the overall area in contact was not higher than 33% of the total surface, and the contact area in the stem was concentrated for both configurations around the stem tip.

### 3.2 Contact sliding micromotions

**FIGURE 5:**
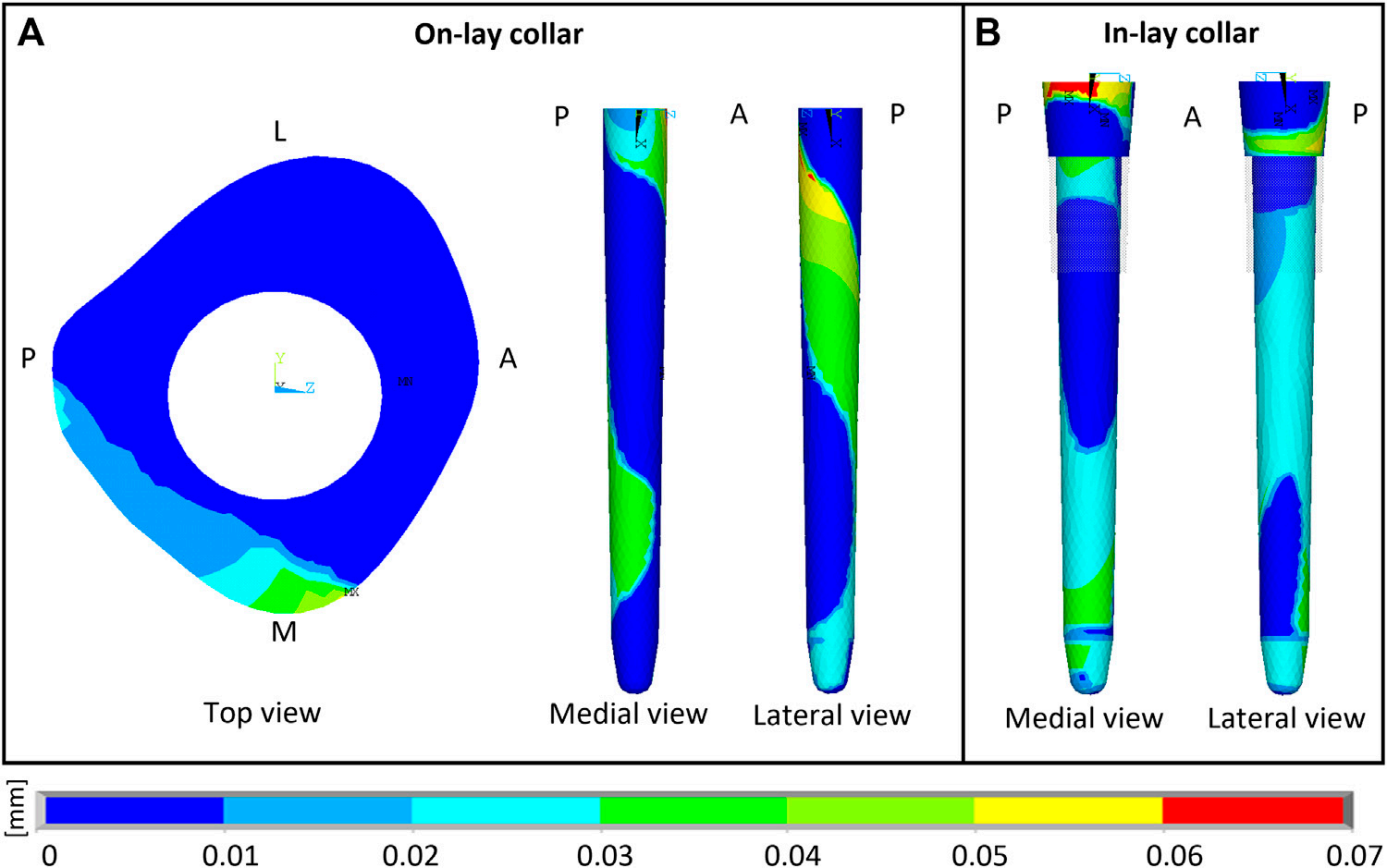
Sliding micromotion resulting from the contact simulation of M-PFR in On-lay **(A)** and In-lay **(B)** collar configurations. A, P, M, L, superscripts indicate Anterior, Posterior, Medial and Lateral aspects, respectively.

Contact sliding micromotions results are summarised in Table 3 and Figure 5. In the P-PFR and M-PFR models of the on-lay configuration, the small collar ring area in contact with bone in the medial and posterior aspect of the osteotomy experienced limited sliding, which was however concentrated in a medial spot, raising a concern for the development of high shear strains nearby (Section 3.3). The stem was subjected to a larger sliding that locally, in the proximal portion, reached values slightly higher than the 50 µm risk threshold for cemented interfaces. P-PFR and M-PFR models of the in-lay configurations showed a smoother contact interaction, with no regions exceeding alert values and a more uniform distribution of micromotions over the stem, likely thanks to the larger area in contact.

D-PFR models showed not alerting levels of micromotions all over the interfaces, both for the in-lay and on-lay configurations. This behaviour, combined with the contact status results, likely indicates that load transfer to bone in D-PFR happens mostly through the stem at its tip.

### 3.3 PFR stresses

The modular PFR assembly relying on relatively short taper junctions helped reducing the PFR stiffness with respect to a monolithic construct, so that flexibility in the coronal plane was maintained, although grossly halved with respect to the intact bone. As expected, peak stresses occurred at the taper junction surfaces and at the distal collar-bone interface, but were maintained at relatively low levels. In all configurations, the maximum nodal Von Mises stress was around 290 MPa at the distal end of the collar-bone interface, which has the narrowest resisting section. This value is well below the fatigue limit we assumed for the titanium alloy the PFR is made of. However, as i) the maximum value arose in a small location close to an edge and ii) we neither modelled the fine details of the geometry (e.g., fillets, which may decrease stress concentration) nor the trabecular titanium surface (which may instead increase stress concentration especially if residual stresses are not relieved), the final conclusions about prosthesis safety should be left to experimental fatigue testing.

### 3.4 Bone strains

For all PFR configurations, the superficial strain field in the distal portion of the femur was almost equal to that of the intact bone. The normal stress distribution due to medio-lateral bending, developing postero-medial compressive and antero-lateral tensile strains was maintained, apart from a few unloaded millimeters distal to the osteotomy in the in-lay configuration (see longitudinal strain maps in Supplementary Appendix S1) within the physiological range of ±0.3% (Burr et al., 1996), where instead a significant, but still physiological hoop strain arose, more evidently for the in-lay design in the P-PFR and M-PFR configurations (see hoop strain maps in Supplementary Appendix S1). Differences in strain with respect to intact conditions were localized at the inner bone-PFR interface, being more marked in the collar-bone interface and levelling off moving distally (longitudinal strain maps in Supplementary Appendix S1). Differences between in-lay and on-lay collar configurations could be also observed when looking at longitudinal, hoop or principal strains at sections corresponding to the collar-bone interface (Supplementary Appendix S1). Principal strains never exceeded damage thresholds, although some concern was raised by unusual strain peaks in the medial part of the on-lay collar-bone interface. In fact, consequently to the sliding-sticking behaviour in the small area of the osteotomy in contact with the on-lay collar, a compressive peak exceeding physiological levels appeared in the postero-medial edge of the osteotomy, and a peak due to high shear strain nearby (shear strain at on-lay collar interface in Supplementary Appendix S1, see also Section 3.3).

### 3.5 Remodelling tendency

**FIGURE 6:**
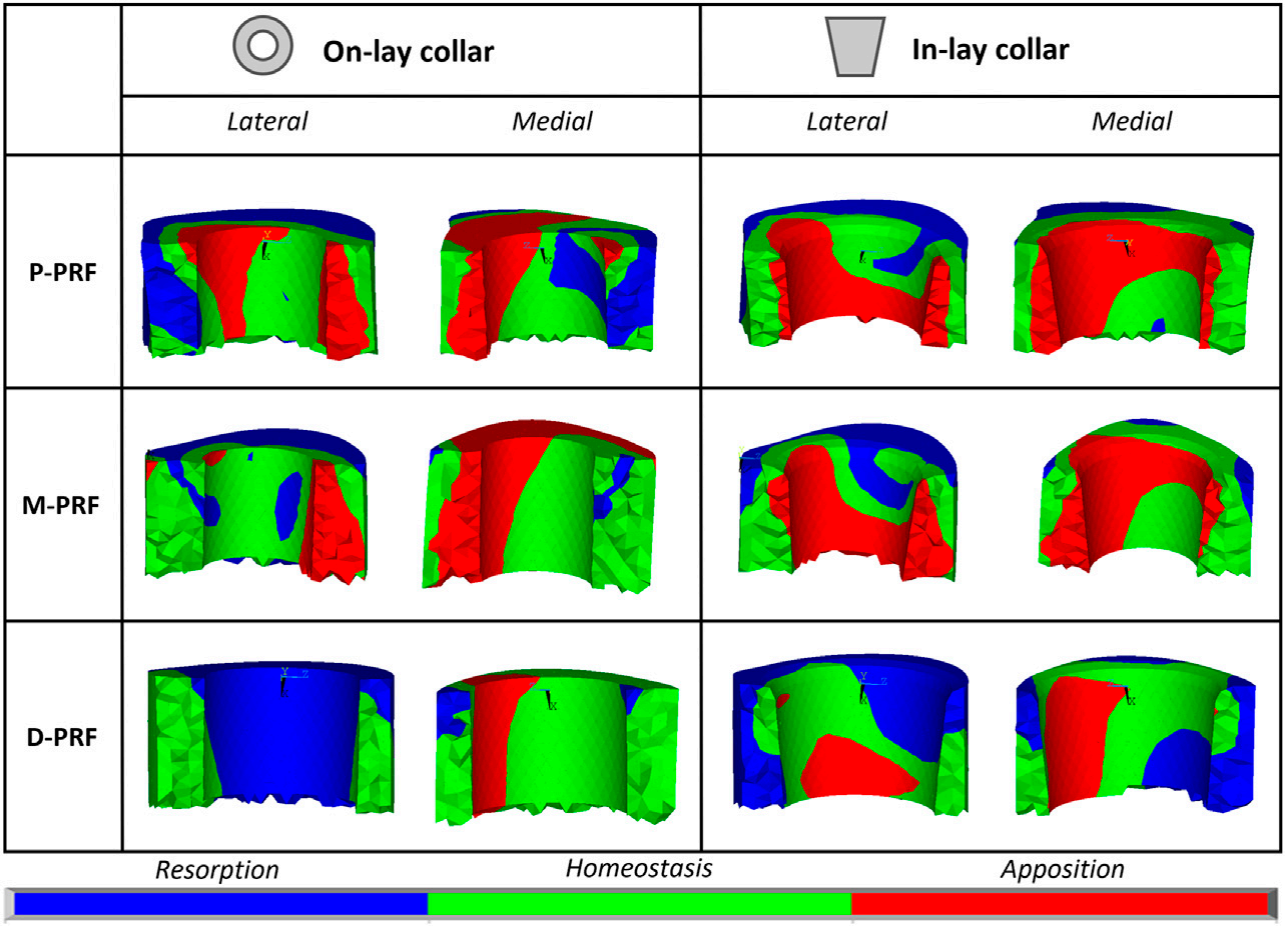
Visual maps of estimated bone resorption, homeostasis or apposition in the volume of interest around (In-lay) or just below (On-lay) the collar region.

Remodelling tendency results are summarised in Table 4 and Figure 6. For all configurations, deviations from homeostasis (i.e., the strain energy density field of the intact bone) increased restricting the VOI towards the implant interface.

**TABLE 4:** Estimated percentage volumes of bone around (In-lay) or just below (On-lay) the collar region that are likely to undergo bone resorption, homeostasis or apposition according to the strain energy density criterion adopted. Results are presented for three regions of interest progressively reducing the volume of interest from the external surface (A) to two (B) or one (C) finite element layers close to the bone-implant interface.

		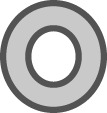 **On-lay collar**			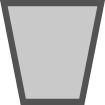 **In-lay collar**			
		*Resorption*	*Homeostasis*	*Apposition*	*Resorption*	*Homeostasis*	*Apposition*	
**P-PFR**	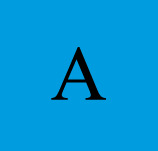	38	50	12	23	56	21	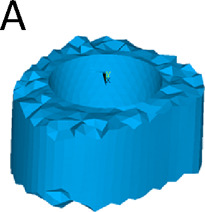
	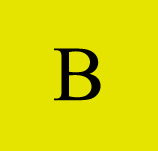	24	54	22	7	44	49	
	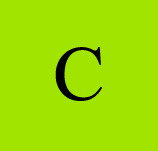	19	48	33	5	27	68	
**M-PFR**	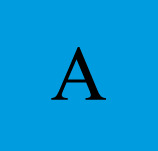	32	47	21	22	56	22	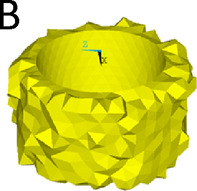
	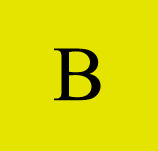	24	53	23	7	49	44	
	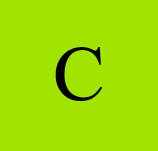	14	60	26	7	32	61	
**D-PFR**	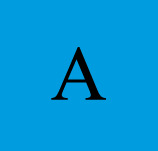	47	48	5	53	38	9	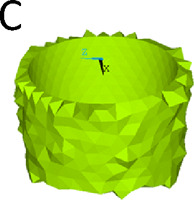
	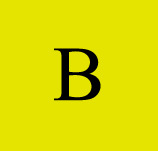	40	51	9	35	50	15	
	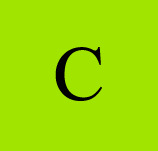	38	50	12	28	47	25	

In the P-PFR and M-PFR, in-lay configurations consistently showed higher (roughly double) volume percentages of predicted bone apposition and reduced (up to one-third) percentages of predicted bone resorption. Predicted appositional behaviour was predominant in the in-lay models, where a few millimetres distal to the osteotomy might undergo resorption but moving distally bone apposition is likely to happen almost all around the interface with the collar sleeve. On-lay models VOIs showed similar percentages of predicted apposition and resorption but indicated possible generalised resorption due to stress-shielding in the outermost lateral aspect.

D-PFR showed generally less favourable maps of the bone remodelling tendency. Overall numbers were similar for in-lay and on-lay configurations. Half of the whole VOI around the sleeve collar was predicted at risk of resorption. Moving closer to the bone-implant interface, this risk tended to decrease. However, the in-lay design seemed to offer a slight advantage also in this case, as the decrease of resorption risk was more marked, and more uniform around the collar interface, while the whole lateral aspect in the on-lay design remained at risk of resorption.

## 4 Discussion

It has previously been demonstrated that osteointegration with extracortical bone-bridging to the implant collar of EPRs improves prosthesis fixation, especially when HA coated collars are used (Chao et al., 2004; Myers et al., 2007). When bone is osseointegrated onto the collar of the prosthesis, survival at 10 years is increased by more than 20% (Coathup et al., 2013).

In this study we investigated the mechanical environment around a proximal femoral (endoprosthetic) replacement (PFR) for two different designs of collar using FE analysis: a novel fully porous bridging collar with a combined endosteal sleeve (in-lay) and a conventional collar without an endosteal sleeve (on-lay). The study examined different reconstruction lengths with the PFR and compared the mechanical environment between the two collar types.

There have been no reported studies investigating the overall mechanical environment around the stem and collar using a complete proximal femur constructed into a validated femur model. The present study used a finite element model to compare a novel in-lay collar to a conventional on-lay collar, in three different conditions for a proximal femoral (endoprosthetic) replacement (PFR) with proximal, mid-diaphysis and distal reconstructions.

We have demonstrated that an in-lay collar creates an advantageous mechanical environment at the bone-collar interface, compared to an on-lay design. The contact area around the collar was significantly increased for the in-lay collar compared to the on-lay collar (60% vs. 40%). The in-lay collar demonstrated more uniform load transfer into the bone with a more physiological pattern, and the predictions of bone apposition were double than with an on-lay collar. The in-lay collar induced a significant hoop strain in the bone cortex, but estimated strain levels remain within the physiological limit, suggesting a condition similar to conventional uncemented stems. Lastly, resorption was reduced by a third utilising an in-lay collar. The main differences in the mechanical environment induced by the on-lay and in-lay designs were found at the collar region, and more evident close to the interface between the bone and collar. Although neither of the designs realise full contact around the collar due to the high stiffness of the overall reconstruction, the in-lay collar may stimulate apposition of bone all around it, while results for the on-lay collar were more polarised.

The on-lay collar suffered significantly greater stem-cement micromotions, non-physiological localised shear and compressive strains at the bone-implant interface and a much larger volume at risk of resorption. These findings may indicate increased risk of prosthetic fractures clinically.

The greatest differences between the in-lay and on-lay designs were identified in the proximal and mid-diaphyseal reconstructions. In more distal reconstructions, results for on-lay and in-lay designs were similar, and overall, less favourable to positive bone remodelling around the implant. Unwin et al. theorised that longer resection in PFRs was protective as the mechanical offset decreases with greater resection length, which may explain the reduced stresses in the distal femur (Unwin et al., 1996).

Few studies have investigated the mechanical environment surrounding the collar and stem in endoprosthetic reconstruction. Fromme et al. (2017) used a finite element model to describe load transfer at the collar-bone interface and confirmed that the stem-collar junction is the region subjected to the highest stresses and crucially that the stresses are greatest when there was no ECBB onto the collar; the authors concluded that this would promote stem fracture under normal walking loads. They showed that bone ongrowth reduced stresses due to more physiological loading thus protecting against implant failure.

The results from this study would indicate that an in-lay collar design would be more likely to achieve ECBB at the collar of the implant and could potentially reduce the likelihood of aseptic loosening. All conventional collars are the on-lay design, and aseptic loosening rates for these devices are reportedly high. Although there are no published reports for the in-lay design, two of the authors of this study have been using the in-lay design for the last 5 years and to date and have observed zero cases of aseptic loosening in a series of over 100 PFRs. This study in combination with excellent short-term clinical data would suggest that a fully porous in-lay collar design could significantly increase the survivorship of endo-prosthetic replacements.

There are several limitations to this study.

A single femur instance was modelled with one load condition. However, the verified mean geometry and clinically relevant density distribution, with a single load condition is a useful way of investigating the relative comparison between the in-lay and on-lay design.

Only cemented fixation was modelled in this study, and although we would like to extend the analysis to cementless fixation, the use of cement is very common with proximal femoral (endoprosthetic) replacements. In the virtual implantation process, we assumed a perfect match between implant and bone; and we did not model a possible press-fit due to the insertion process of the collar in the reamed cavity. Modelling these surgical conditions and variabilities was out of the scope of the study and would have required modelling of inelastic phenomena, for which the modelling procedure was not verified and validated. A direct validation against experimental data for the studied prosthetic configuration could not be produced. We however used a modelling procedure that has been extensively validated in the estimate of bone strains, bone failure, and bone-implant interaction (although in a different femoral prosthesis design). This same procedure had also been used previously to successfully revise an implant design (Martelli et al., 2011a).

The validated modelling procedure adopted neglects bone anisotropy, while the mechanical response of cortical bone of the femoral diaphysis, where the studied reconstructions insist on, is acknowledged to be anisotropic. To verify the strong assumption of bone isotropy we developed an additional anisotropic model of the intact bone and of the mid-diaphyseal reconstruction, computing bone strain and strain energy metrics. This additional activity, fully reported in Supplementary Appendix S2, highlighted: i) the limited robustness of anisotropic material parameters, and ii) the limited changes in the classification of the risk of bone damage in exercise and of bone weakening over time when anisotropic material properties were introduced (to our best estimate) in the model. We therefore corroborated the use of a validated isotropic model, even though the development of a validated anisotropic model would be desirable to further increase the accuracy and biofidelity of the analysis.

We did not develop or implement a model of the bone remodelling process. However, validated bone remodelling models for human bones from continuum level imaging data are not available, to our knowledge. Validated models are available only from microCT images in specific mice breeds, which have limited genetic (and age) variability, contrarily to humans. Therefore, we preferred limiting to give an indication of a potential remodelling signal based on strain energy density trends.

Finally, the porosity of the trabecular titanium layer of the in-lay collar was not modelled explicitly, but indirectly through a higher coefficient of friction with the surrounding bone. Future research could model in more detail ingrowth throughout the porous collar. A more porous structure might permit swifter ingrowth of bone directly from the transected cortex thereby improving osseointegration (Bram et al., 2006) as has been shown in animal modelling (Mumith et al., 2017).

In summary, the novel design in-lay collar provides an advantageous mechanical environment at the resection level compared to the existing on-lay design, with approximately twice as much bony apposition and significantly lower bony resorption. This has clear potential benefits in the clinical setting.

## Data Availability

The datasets presented in this article are not readily available because the implant models were generated using proprietary designs of Adler Ortho S.p.A. Request to access these data should be addressed to info@adlerortho.com. Access to the anonymised images used to build the bone models is available by request to the corresponding author. Requests to access the datasets should be directed to ES, enrico.schileo@ior.it.
