# Supplementary material for: Does a novel bridging collar in endoprosthetic replacement optimise the mechanical environment for osseointegration? A finite element study
Source: Front Bioeng Biotechnol. 2023 Jun 5;11:1120430. doi: 10.3389/fbioe.2023.1120430 (PMC10277679; doi:10.3389/fbioe.2023.1120430)
Supplement: Supplementary file 1 [file DataSheet2.PDF]

APPENDIX 2: Anisotropic bone modelling

In response to a comment from one Reviewer, and considering the acknowledged anisotropic nature of femoral cortical bone, we applied an anisotropic material model (transverse isotropy) to the intact and in-lay collar configurations, computing strains and strain energy densities and comparing them with isotropic models.

Foreword

For the isotropic model we could rely on a validated FE modelling procedure, that uses a site-specific (femoral) density-elasticity relationship derived from state of the art mechanical tests (for a complete rationale on this choice please see Helgason et al, Clin Biomech 2008, 23(2):135-46; and Schileo et al., J Biomech 2007, 40(13):2982-9.). Results of this FE modelling procedure have shown to accurately reproduce experimental strain measurements in different conditions, at different locations including the diaphysis (Schileo et al., J Biomech 2008, 41(11):2483-91) and also in implanted conditions (Taddei et al., J Biomech Eng. 2010 Feb;132(2):021002)

This was not the case for anisotropic material modelling. Athough it is clear that cortical bone is anisotropic, we are not aware of an extensive validation of a FE modelling procedure including cortical bone anisotropy at the femur. Conversely, we are well aware of the difficulties in measuring all the (at least five) anisotropic material parameters and in establishing the dependence of their magnitude and direction, if any, on density or on other structural, compositional or organisational parameters.

Definition of the anisotropic material model

We conducted our anisotropic modelling test:

- considering the mid-diaphyseal reconstruction (which insists on the mid-diaphysis, where the majority of anisotropic tests have been carried out, and the shaft is quite aligned to a longitudinal direction);
- assuming transverse isotropy to reduce to a minimum of five the independent elastic constants, also in light of several studies showing that the anisotropy ratio between the principal direction and the other two is significantly larger than that between the two secondary directions
- assuming the longitudinal shaft direction as principal material orientation (the transversal isotropy plane thus including radial and circumferential directions)
- assuming the Elastic modulus in the principal material orientation (longitudinal) equal, for each element, to that of the isotropic model, for consistency with the validated isotropic modelling strategy, and to highlight any effect of anisotropy in the results
- deriving anisotropy ratios by averaging results from different literature works using ultrasound (US) testing to characterise anisotropy (Grimal et al., J Biomech 2011 May 17;44(8):1621-5; Espinoza Orìas et al. J Mech Behav Biomed Mater 2009 Jul;2(3):255-63; Rudy et al., J Biomech. 2011 Jun 3;44(9):1817-20)

Table 1: *transverse isotropy relationships. Axis z indicates the longitudinal direction, x and y identify the transversal plane of isotropy*

TRANSVERSE ISOTROPY RELATIONSHIPS
$E_z = 6850\ \rho_{app}^{1.49}$
$E_x = E_y = 0.666\ E_z$
$G_{xy} = 0.200\ E_z$
$G_{xz} = G_{yz} = 0.286\ E_z$
$\nu_{xy} = 0.23$
$\nu_{xz} = \nu_{yz} = 0.38$

We deemed US tests more reliable either than (i) mechanical tests, that are prone to experimental artefacts if specimens are not included in endcaps and measured multiple times (Helgason et al, Clin Biomech 2008, 23(2):135-46) and are therefore generally not well suited to conduct multiaxial tests; or (ii) indentation-microstructural tests (Reisinger et al., J Mech Behav Biomed Mater. 2011 Nov;4(8):2113-27) that cannot consider architectural aspects beyond the ultrastructure, which are instead present in bone at the continuum level.

Please note that, to more robustly include the effect of anisotropy, the dependence on the longitudinal level along the diaphysis (Espinoza Orìas et al. J Mech Behav Biomed Mater 2009 Jul;2(3):255-63), and on the anatomical quadrant (Li et al., J Mech Behav Biomed Mater. 2013 May;21:109-20) should have been also taken into account. Moreover, while literature somehow converges on the measurement of anisotropy ratios for the longitudinal vs. axial/circumferential direction, this is not the case for the determination of shear moduli and Poisson's ratios. There is recent, preliminary evidence that bone micromechanical models based on an accurate knowledge of microstructure, porosity, mineralization and collagen properties might help accurately characterise the elasticity tensor (Cai et al., Acta Biomater. 2019 May;90:254-266), but knowledge of these parameters would require multimodal ex-vivo imaging and cannot be obtained from images taken at clinical resolution as in our case.

Results

Results for the isotropic and anisotropic models, and changes between the two models, are reported in Figures 1,2 and in Table 2 here below. They can be summarised as follows:

1) The maximum changes occurred, as expected, in the circumferential (hoop) strains. Hoop strain is induced mostly by the presence of the stem and most of all of the collar, thus it is maximum at the endosteal bone surface in close proximity to the collar region of the in-lay (conical sleeve) collar. For this reason, Figure 1 depicts hoop strains around the collar and stem levels, sectioned by a coronal plane to show the anterior and posterior aspects of the endosteal surface.

2) It is notable that the distribution (map) of hoop strains did not change between the isotropic and anisotropic model. The peak located at the proximal edge of the collar sleeve should be taken with some caution and may be overestimated because we could not accurately model the fillet due to defeaturing and mesh resolution.

3) Changes in strains between the anisotropic and the isotropic models are apparent and significant. Hoop strains in the anisotropic models are larger, but the increase is limited to a maximum of 1000 microstrains. Notably, maximum values did not exceed 3000 microstrains (not even in the anisotropic model), which is regarded as a sort of physiological limit in regular exercise (no yellow or red contours visible in Figure 1). Therefore, the inclusion of anisotropic response did not modify our conclusion about the absence of an apparent risk of damage/fracture for bone tissue in the in-lay configuration.

4) We computed and compared the assumed remodelling stimulus (i.e. the change in strain energy density with respect to the intact bone) in the isotropic and anisotropic models, using the same method outlined in the manuscript. Results are summarised in Table 2. Volumes of bone predicted to be in apposition/homeostasis/resorption did not change more than 2% between the isotropic and anisotropic configurations. It is thus very unlikely that the consideration of anisotropy could significantly modify the estimates of bone resorption/apposition tendency over time.

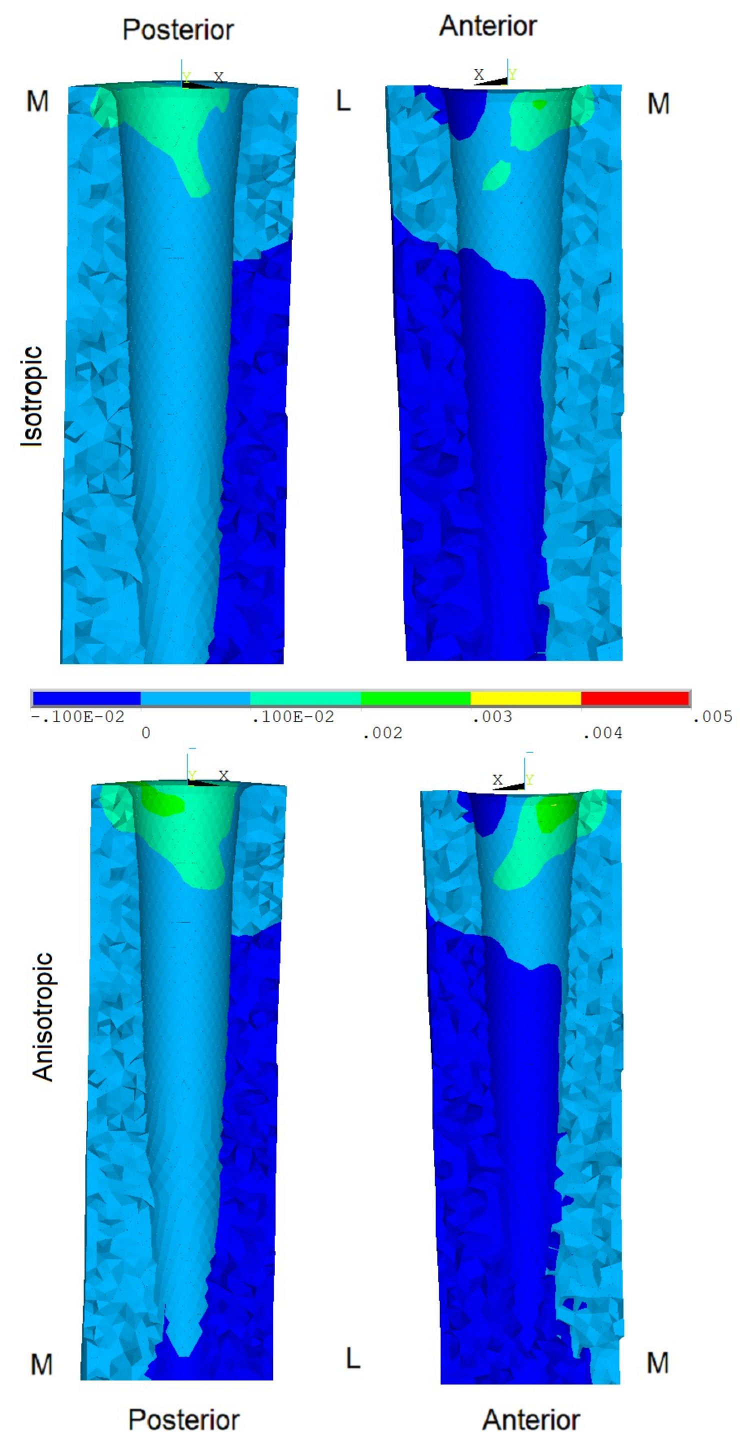

Figure 1: *hoop strains around the collar and stem levels, for the isotropic (top) and anisotropic (bottom) models, sectioned by a coronal plane to show the posterior (left) and anterior (right) aspects of the endosteal surface*

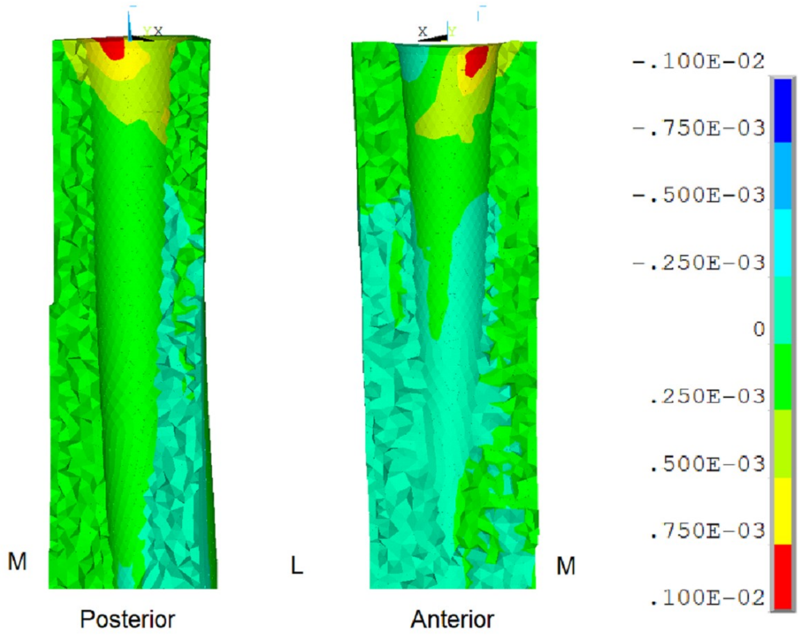

Figure 2: *Changes in hoop strains between the anisotropic and isotropic models*

Table 2: *Estimated percentage volumes of bone around the in-lay collar that are likely to undergo bone resorption, homeostasis or apposition according to the strain energy density criterion adopted. Results are presented for the three regions of interest progressively reducing the volume of interest from the external surface (A) to two (B) or one (C) finite element layers close to the bone-implant interface.*

		**In-lay collar**		
		Resorption	*Homeostasis*	*Apposition*
**Anisotropic**	A	21	55	24
	B	8	47	45
	C	7	30	63
**Isotropic**	A	22	56	22
	B	7	49	44
	C	7	32	61
		A 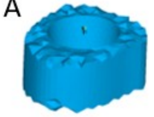	B 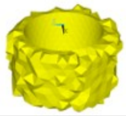	C 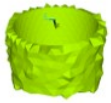

Discussion

In summary, notwithstanding a significant effect of the consideration of bone anisotropy in the development of hoop strains around the collar, it is unlikely that anisotropic model could change either the classification of the risk of bone damage in exercise, or the estimates of the risk of bone weakening over time due to strain-shielding.
