# Supplementary material for: Does a novel bridging collar in endoprosthetic replacement optimise the mechanical environment for osseointegration? A finite element study
Source: Front Bioeng Biotechnol. 2023 Jun 5;11:1120430. doi: 10.3389/fbioe.2023.1120430 (PMC10277679; doi:10.3389/fbioe.2023.1120430)

APPENDIX 1.

This appendix reports full results maps obtained from FE analysis of the PFR femoral reconstruction, to complement the results reported in the main text of the manuscript.

Results from different reconstruction lengths (P-PFR, M-PFR and D-PFR) are presented consecutively, reporting, for each reconstruction length, results from models of the in-lay and on-lay collar design, and comparing results with the intact bone where appropriate.

Results are reported in terms of:

- Contact status

Contact status is mapped to the whole contact interface, according to the classification given by the finite element solver ANSYS

- Contact sliding micromotions

Contact sliding micromotions are mapped to the whole contact interface and scaled to the maximum computed value. Maps of in-plane shear strains are also reported for P-PFR and M-PFR models of the On-lay collar configuration to highlight how the sliding/sticking behaviour in the small area in contact may reflect in the development of non-physiological shear strains at the bone interface

- Longitudinal strains

Longitudinal strains are reported at the bone surface and in extended sagittal sections to show that both collar configurations do not significantly alter the overall strain fields (highlighting the tensile/compressive transition due to medio-lateral bending) found in the intact bone.

- Principal strains

Principal strains (maximum and minimum) are instead reported in the smaller volume of interest around/below the collar to highlihght local changes in strain fields due to the different collar configurations.

- Hoop strains

Circumferential (hoop) strains are reported in extended sagittal sections to exclude that the prosthesis (and the in-lay design in particular) could induce excessive strains in the circumferential direction of the femoral shaft when the stem is mainly subjected to axial compression during gait. Results show that in P-PFR and M-PFR the in-lay design indeed induces higher circumferential strains, that anyway never exceed 3000 microstrains. In D-PFR, coherently with other results, in-lay and on-lay designs show similar results, with lower hoop strain values (< 1000 microstrains).

Proximal-PFR

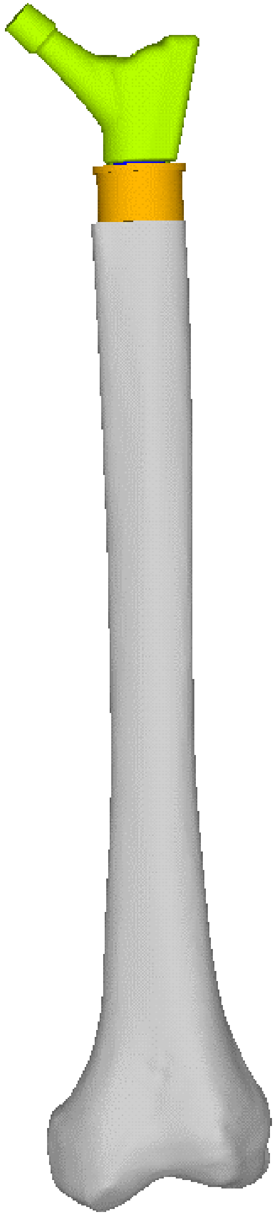

P-PFR / Contact status

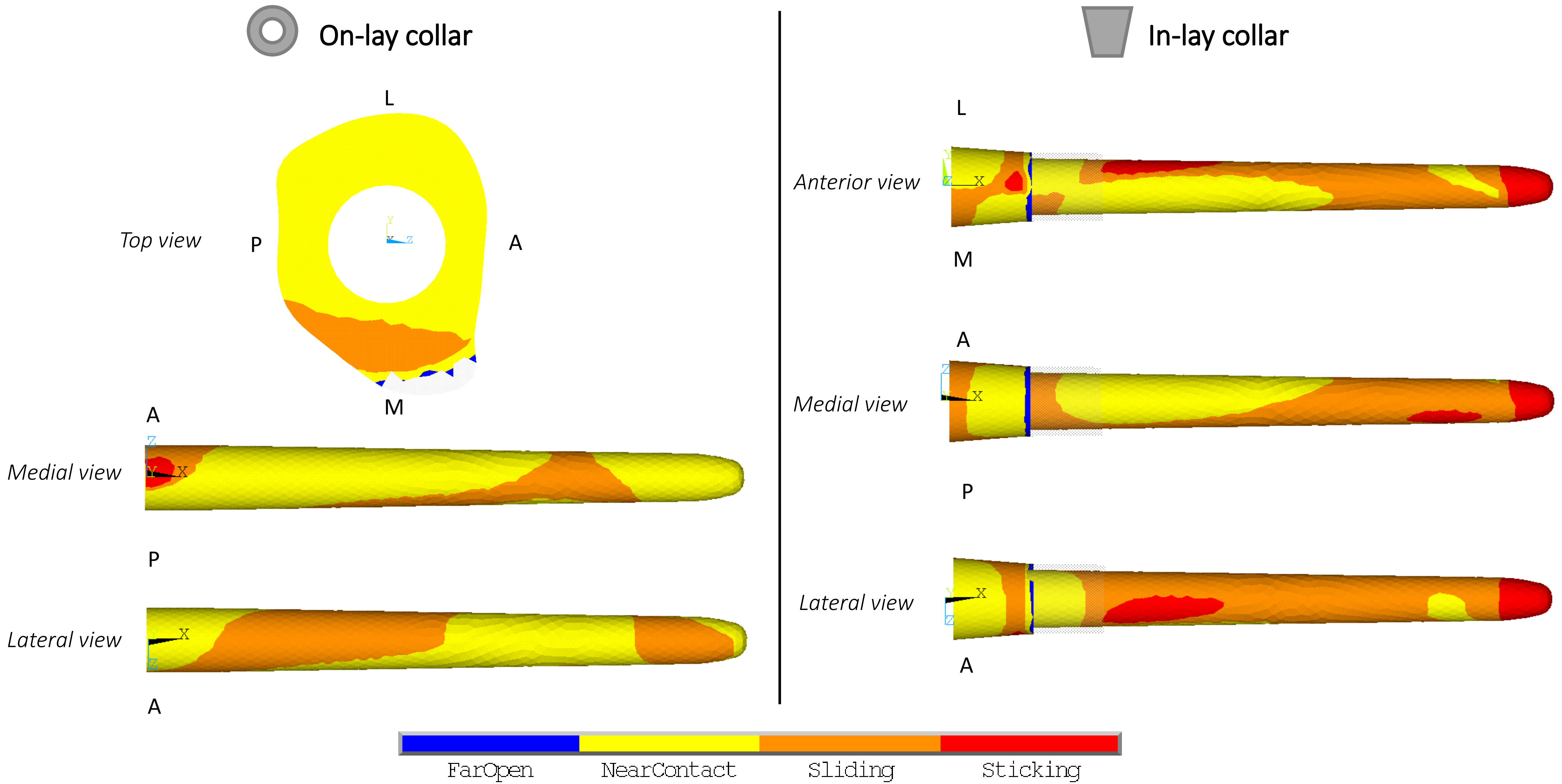

P-PFR /Sliding Micromotions

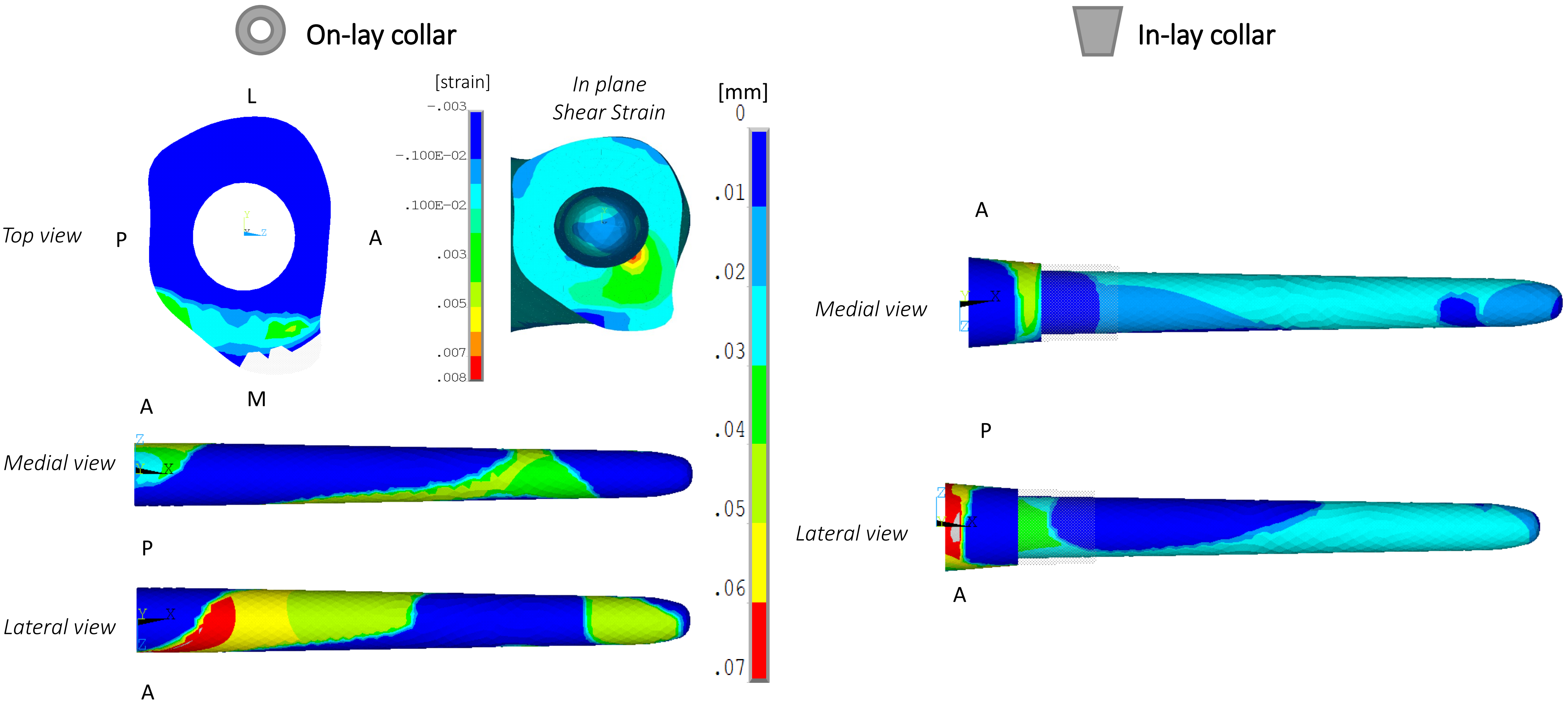

P-PFR /Longitudinal Strain

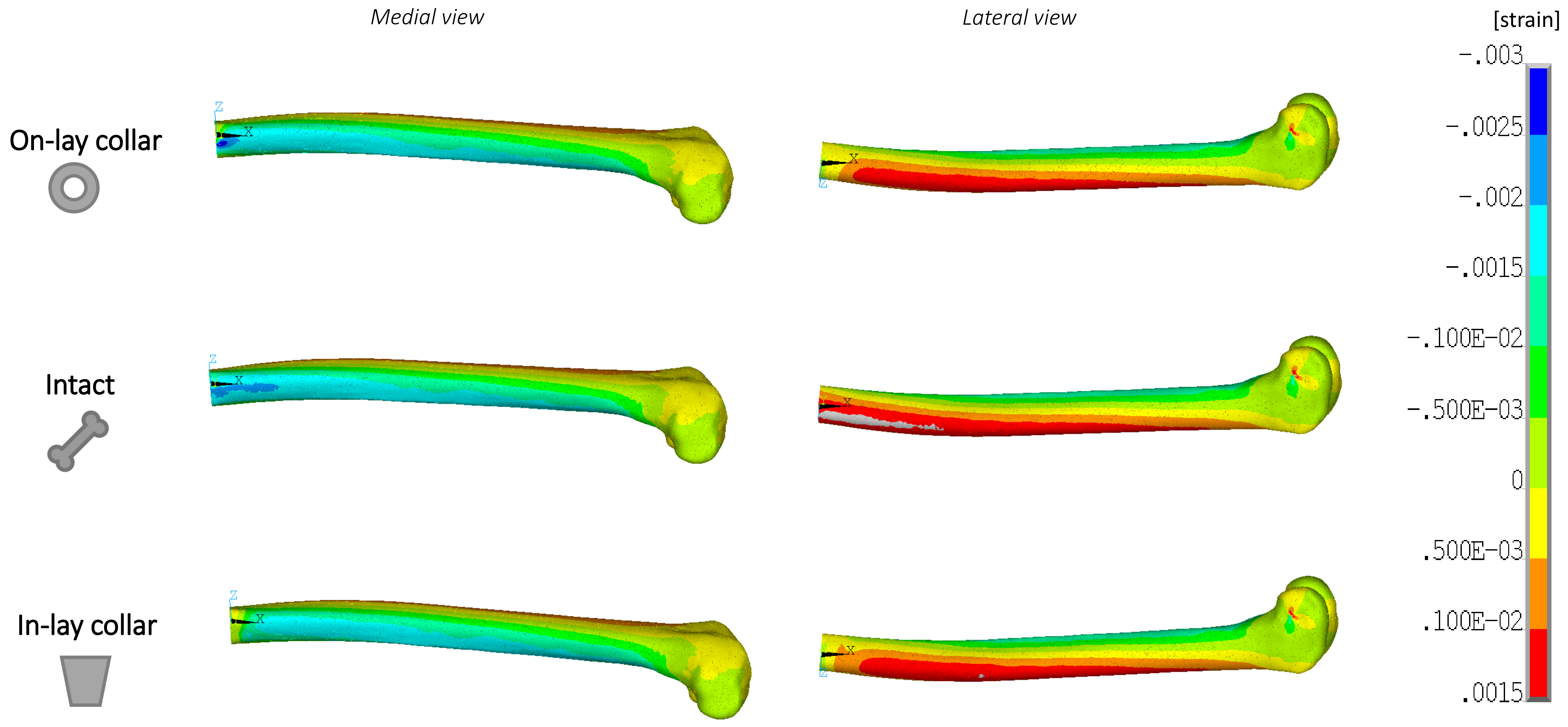

P-PFR /Longitudinal Strain

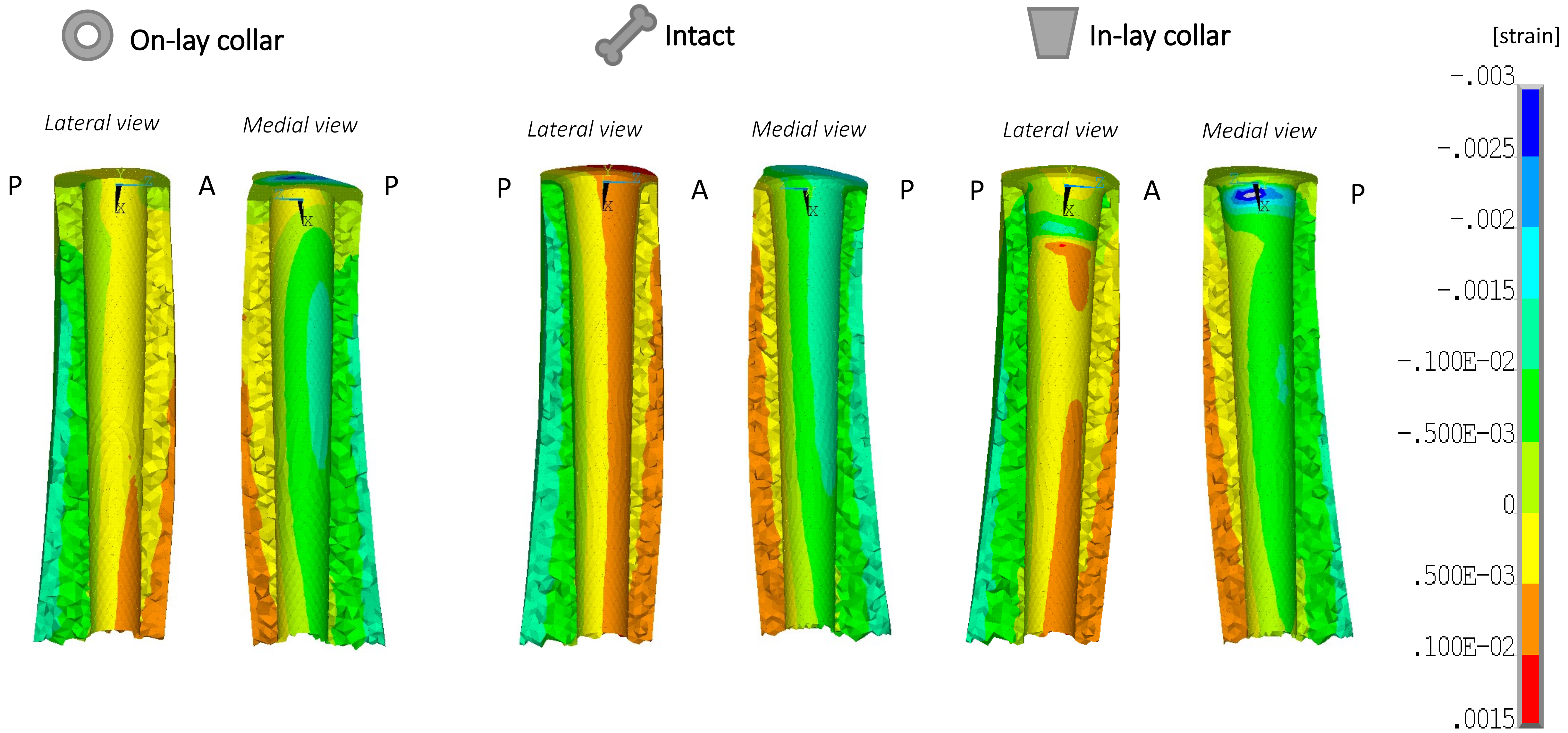

P-PFR /1st (tensile) and 3rd (compressive) Principal Strain

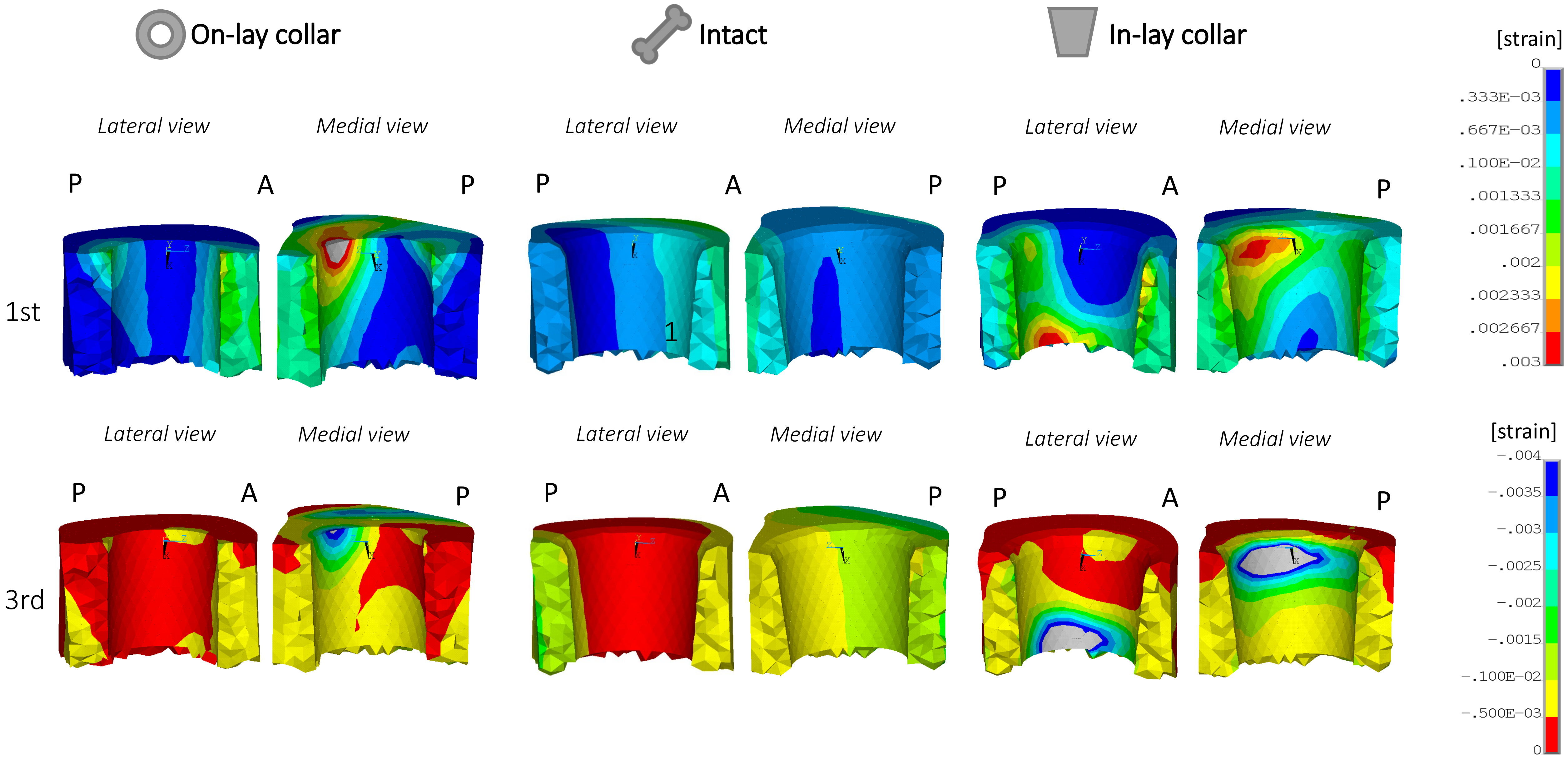

P-PFR / Hoop Strain

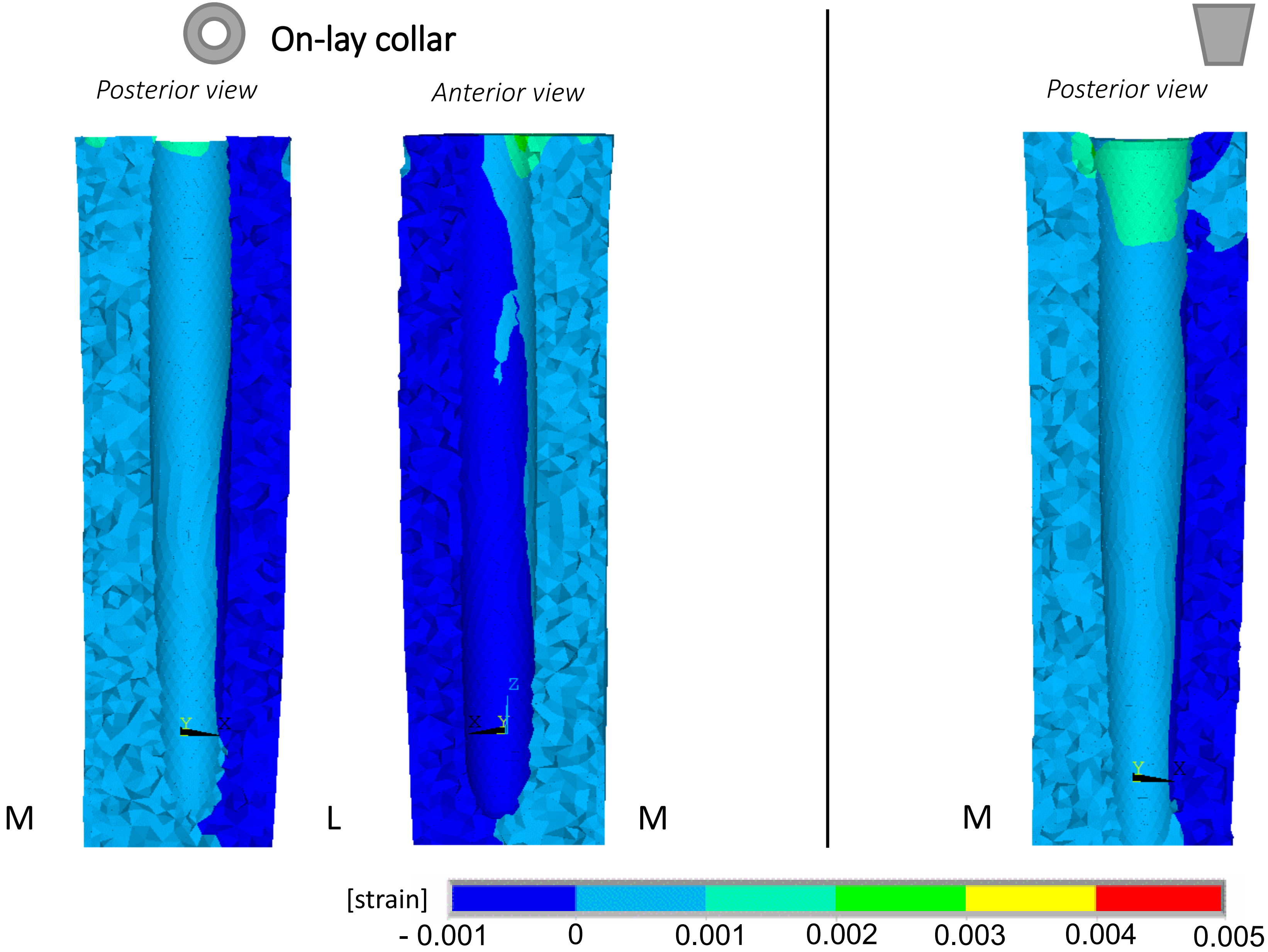

Medial-PFR

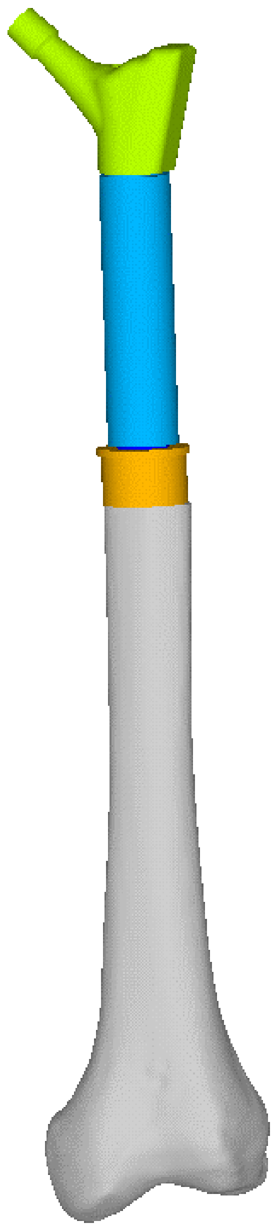

M-PFR / Contact status

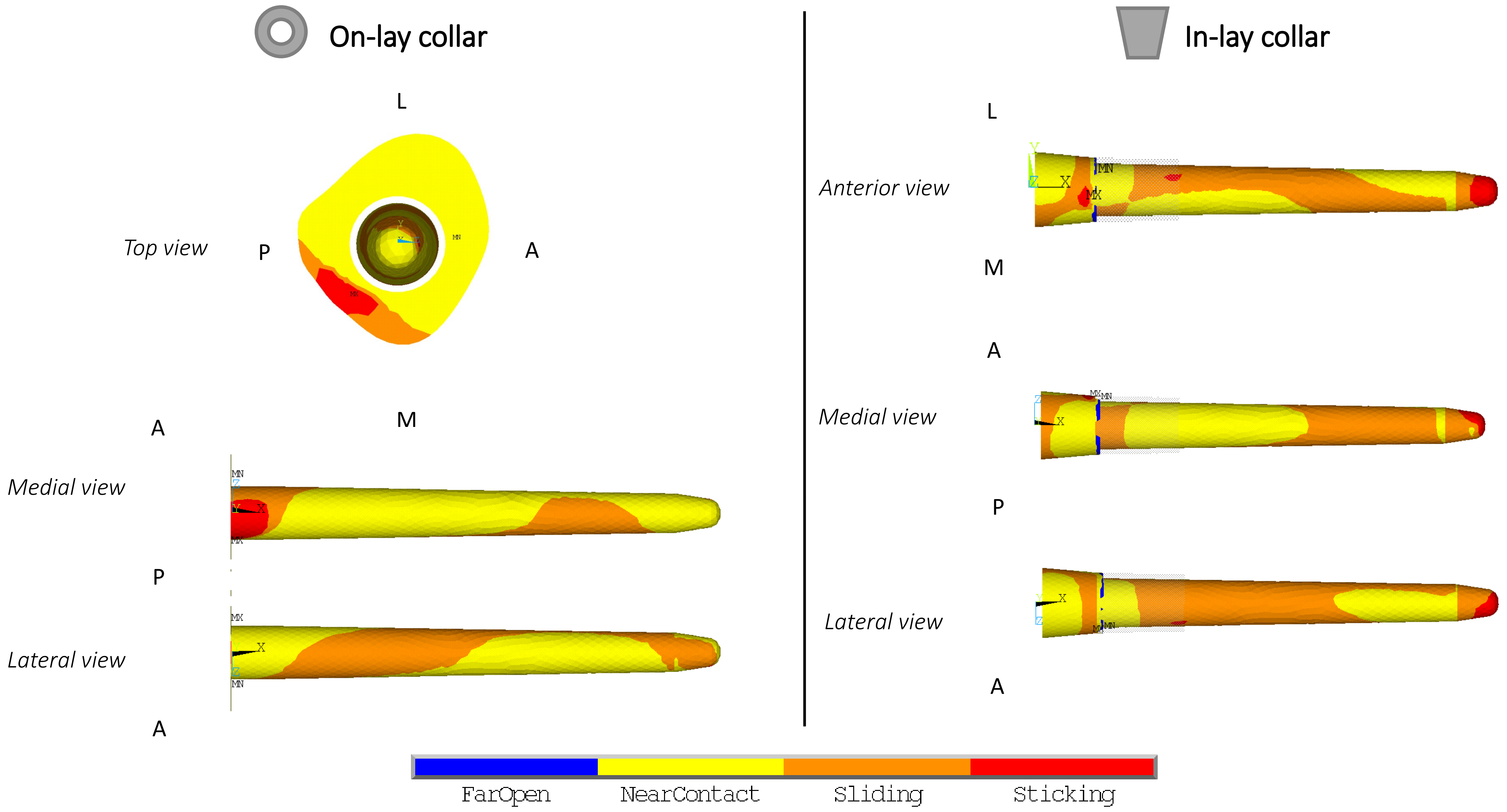

M-PFR /Sliding Micromotions

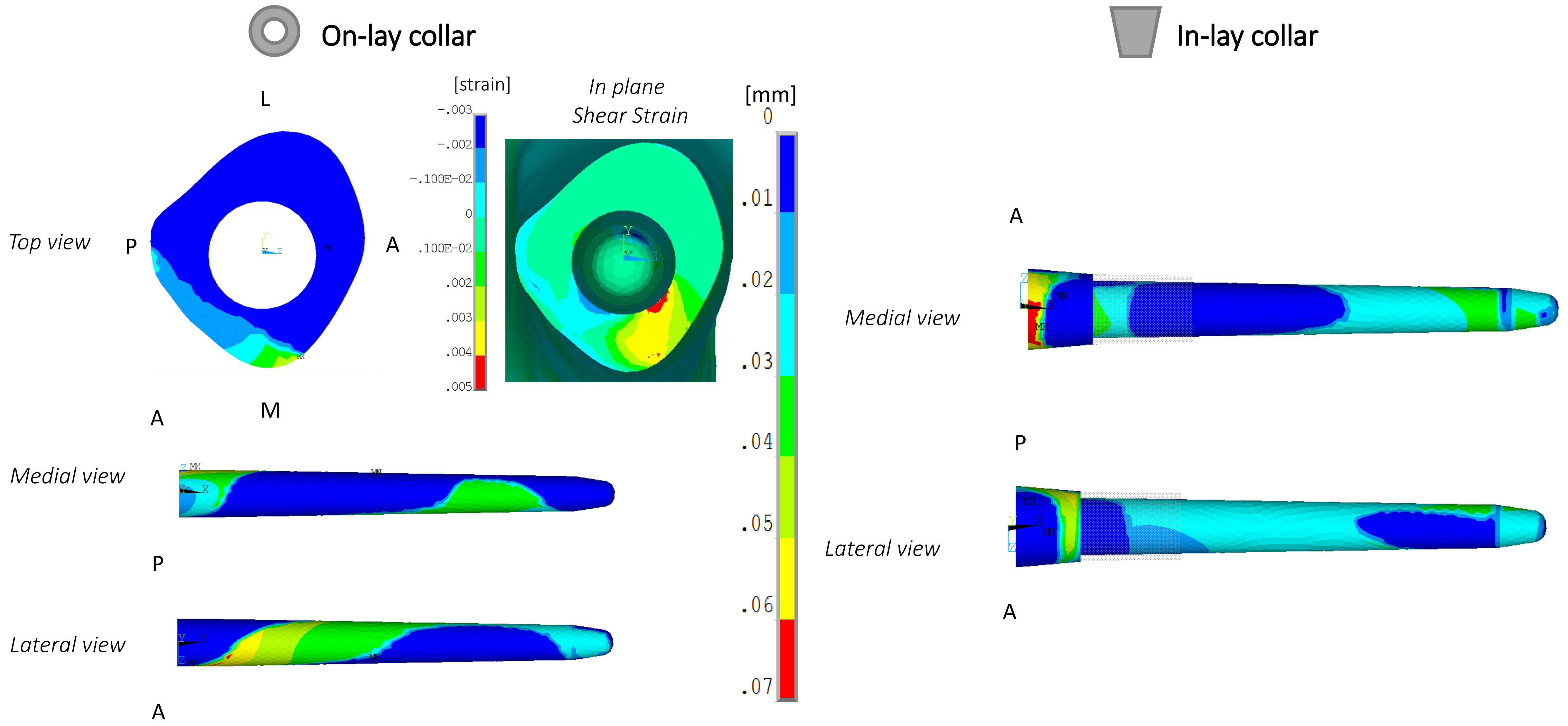

M-PFR /Longitudinal Strain

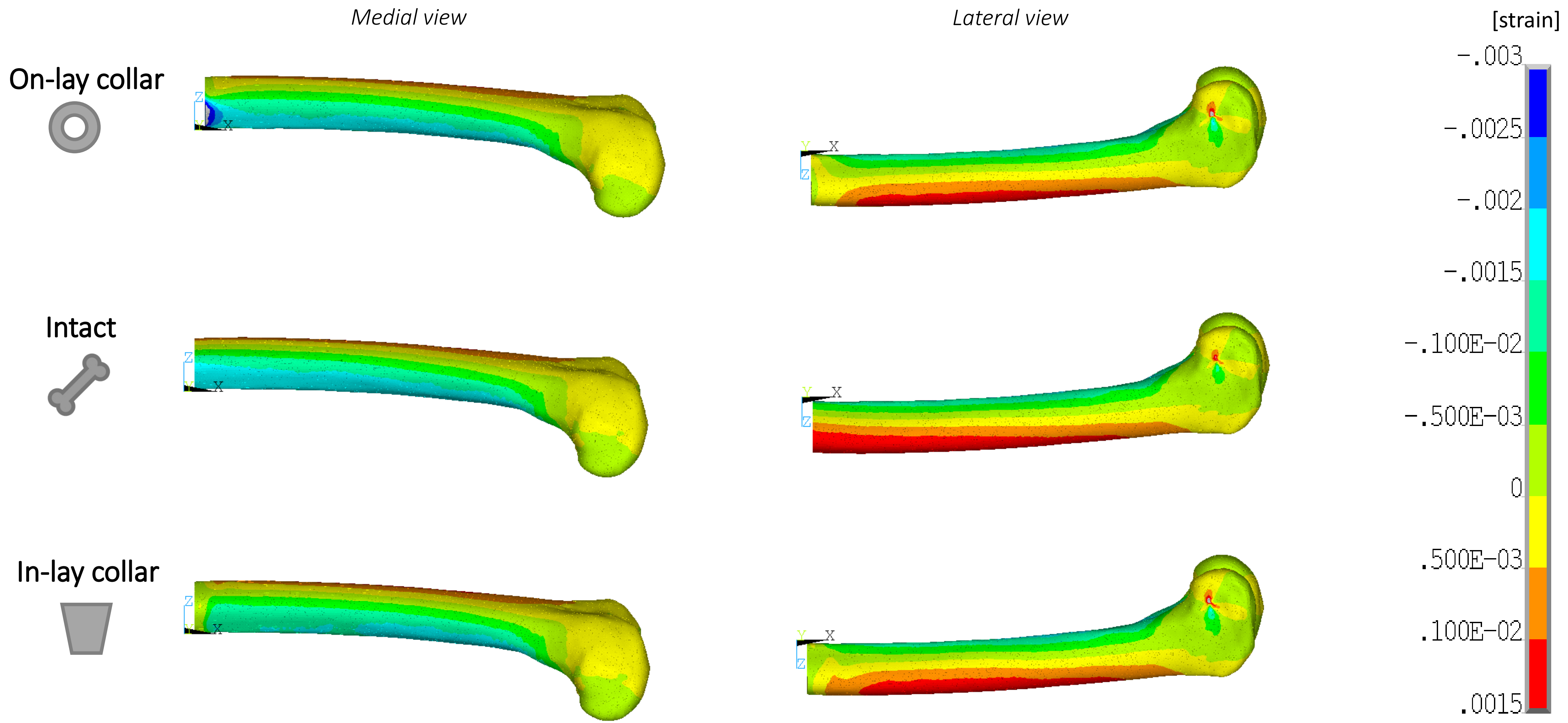

M-PFR /Longitudinal Strain

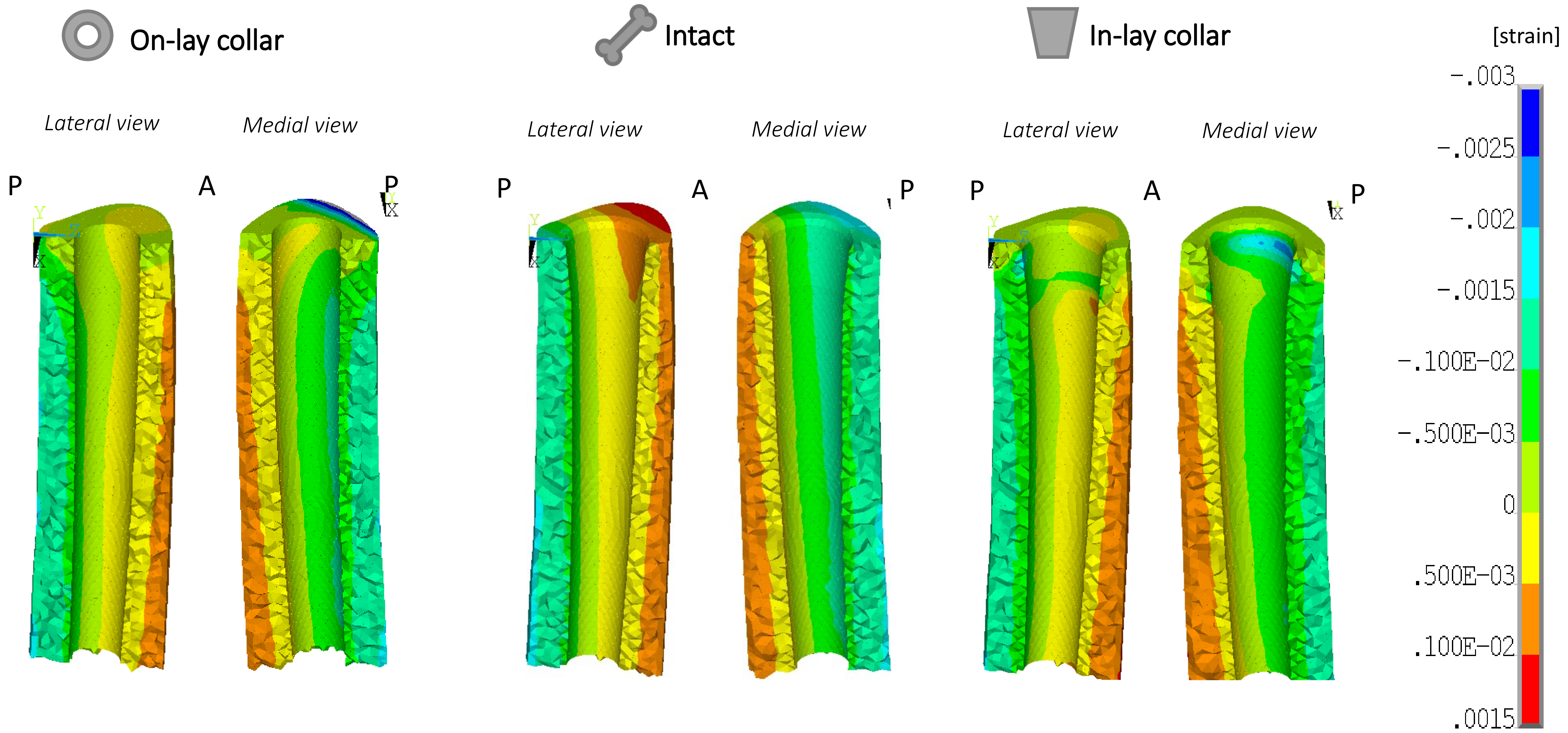

M-PFR /1st (tensile) and 3rd (compressive) Principal Strain

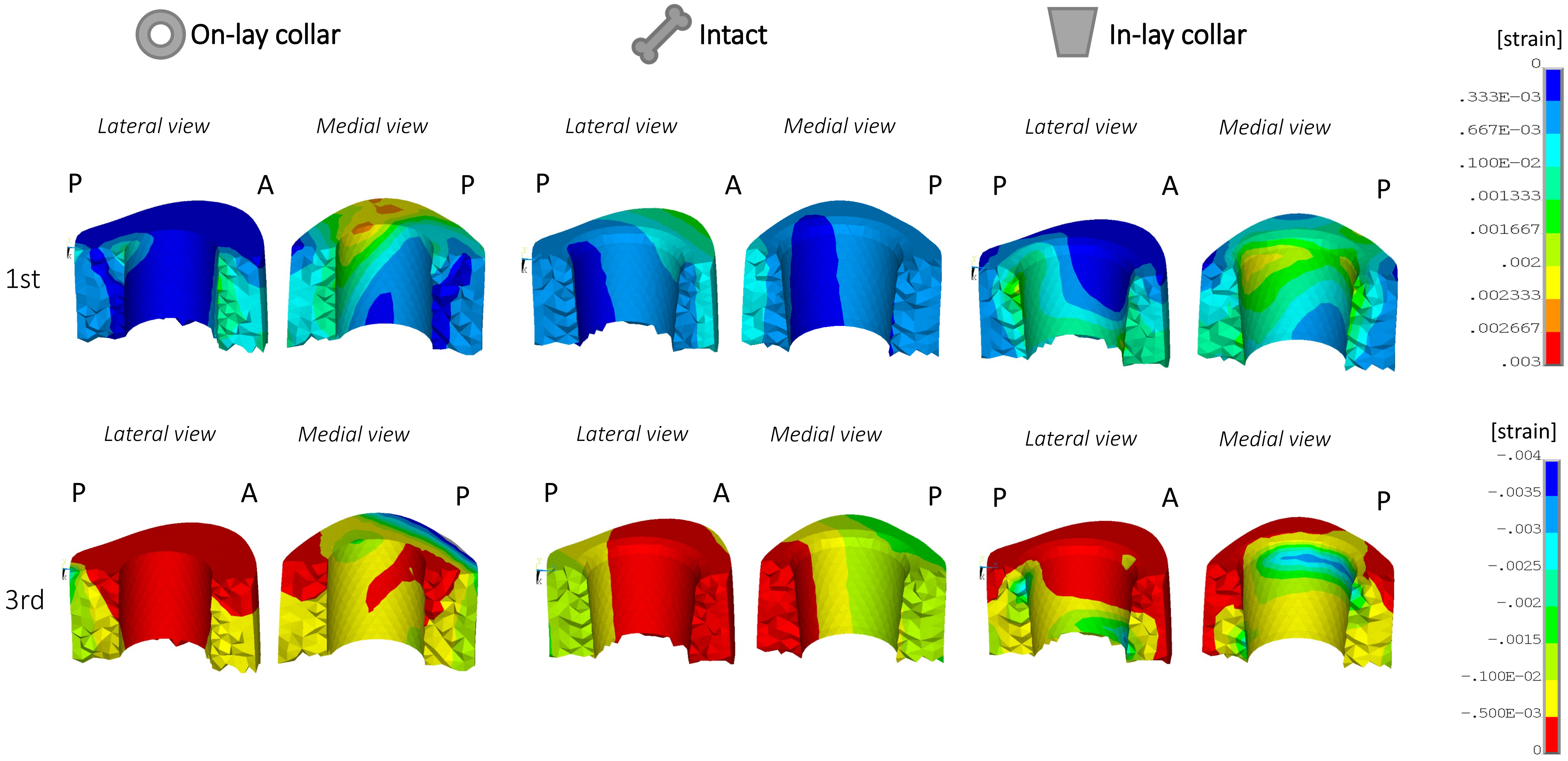

M-PFR / Hoop Strain

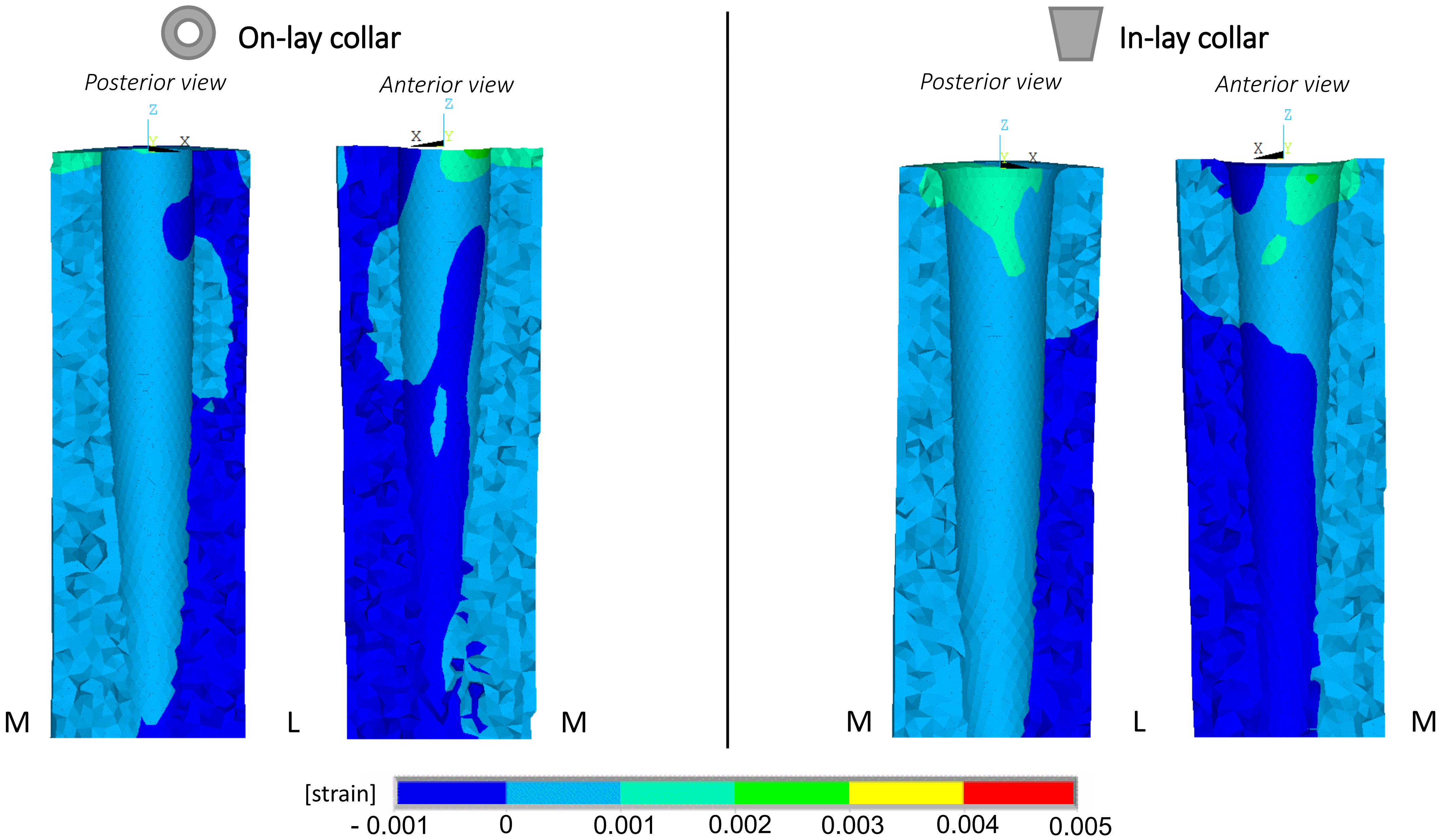

Distal-PFR

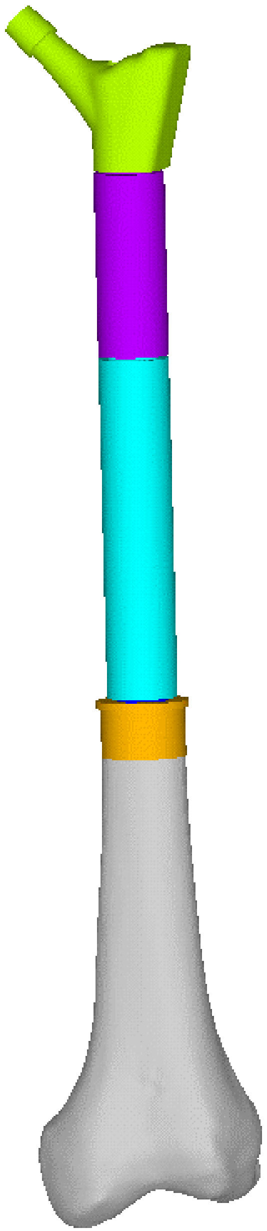

D-PFR / Contact status

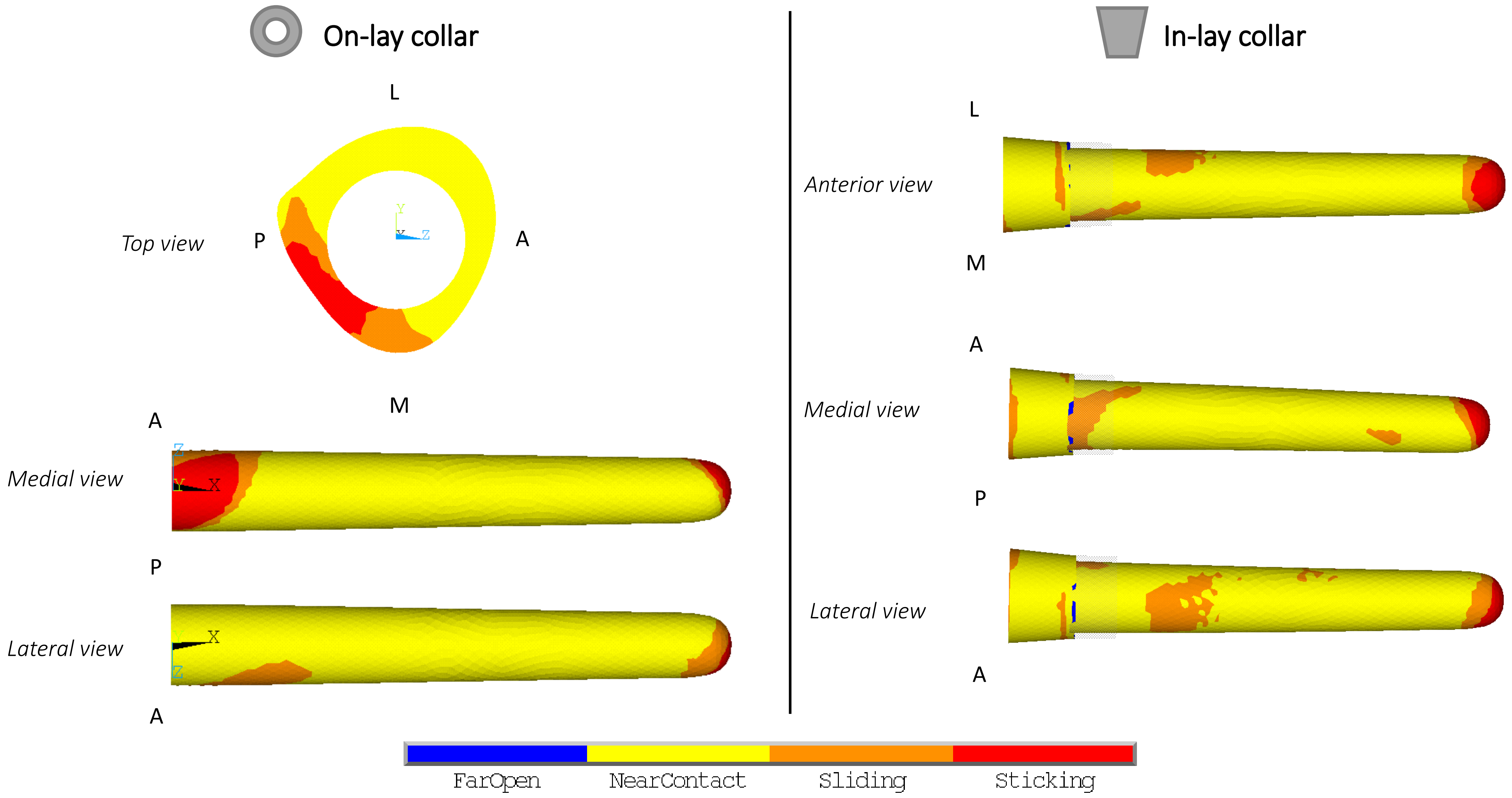

D-PFR /Sliding Micromotions

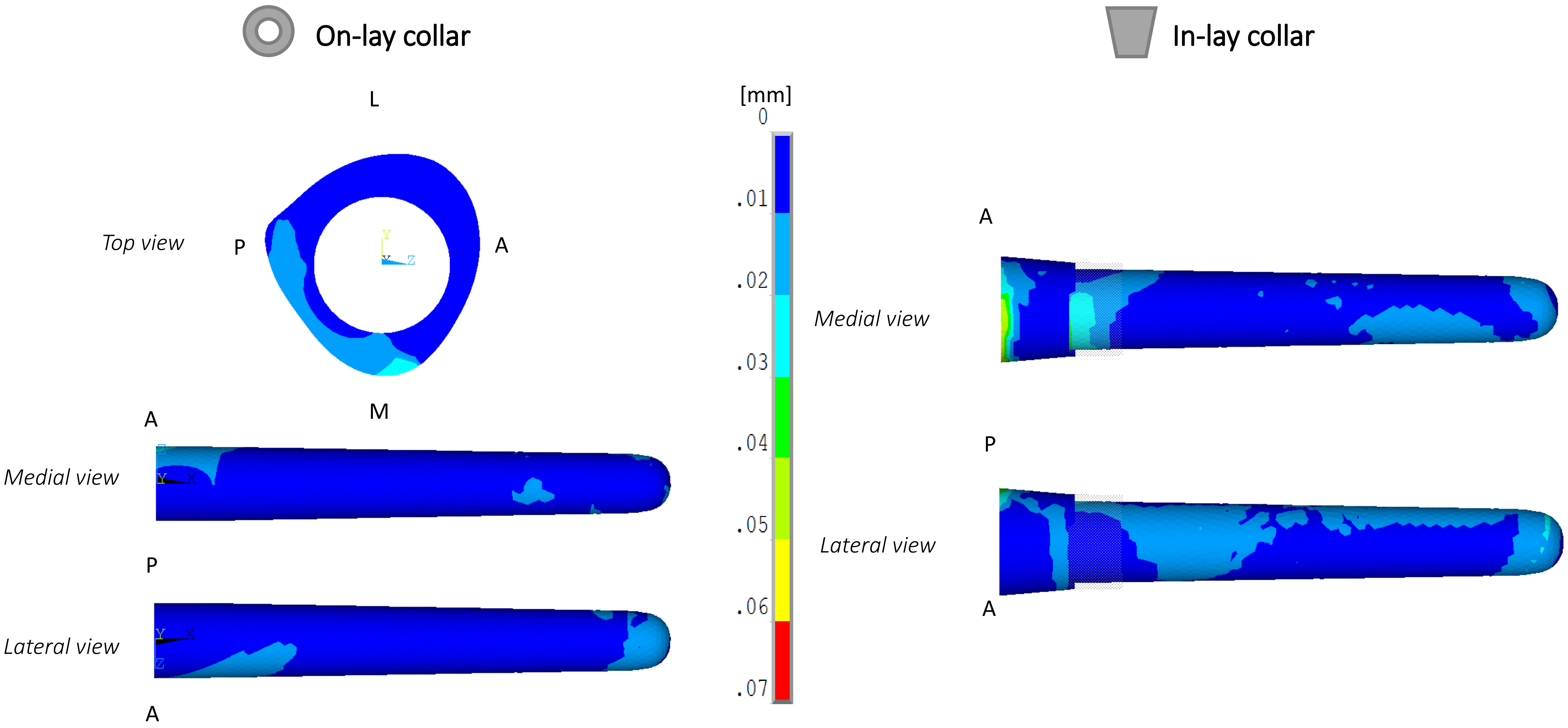

D-PFR /Longitudinal Strain

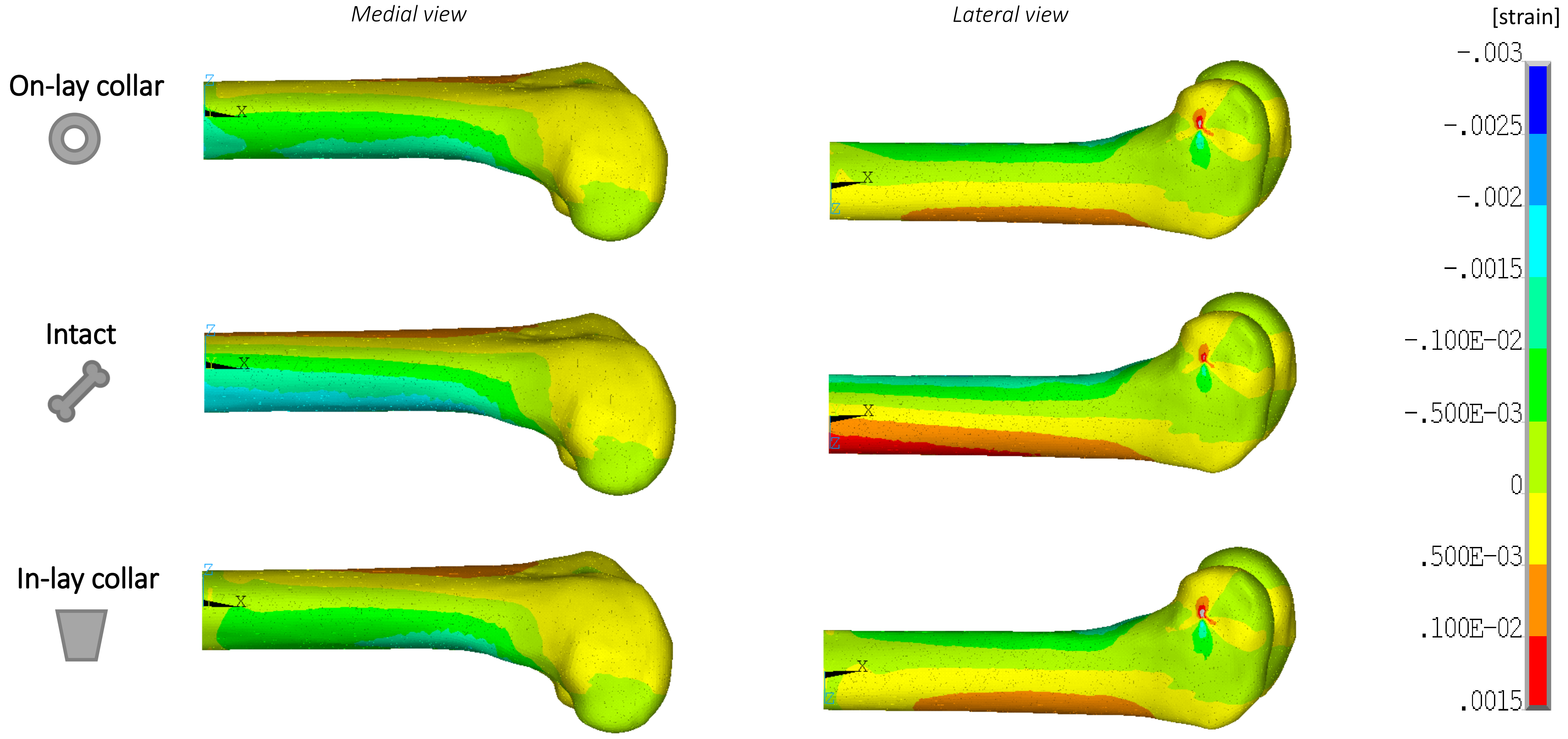

D-PFR /Longitudinal Strain

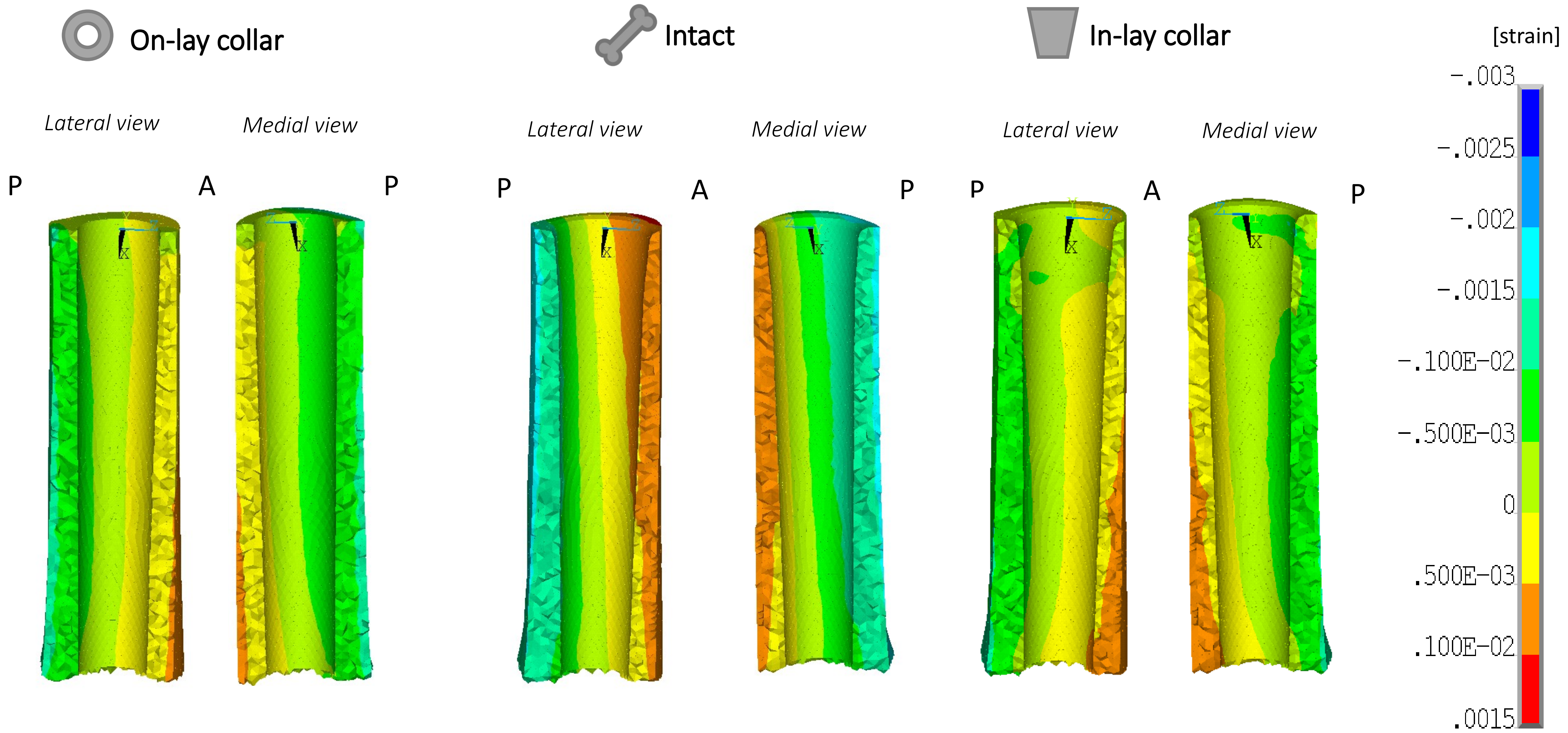

D-PFR /1st (tensile) and 3rd (compressive) Principal Strain

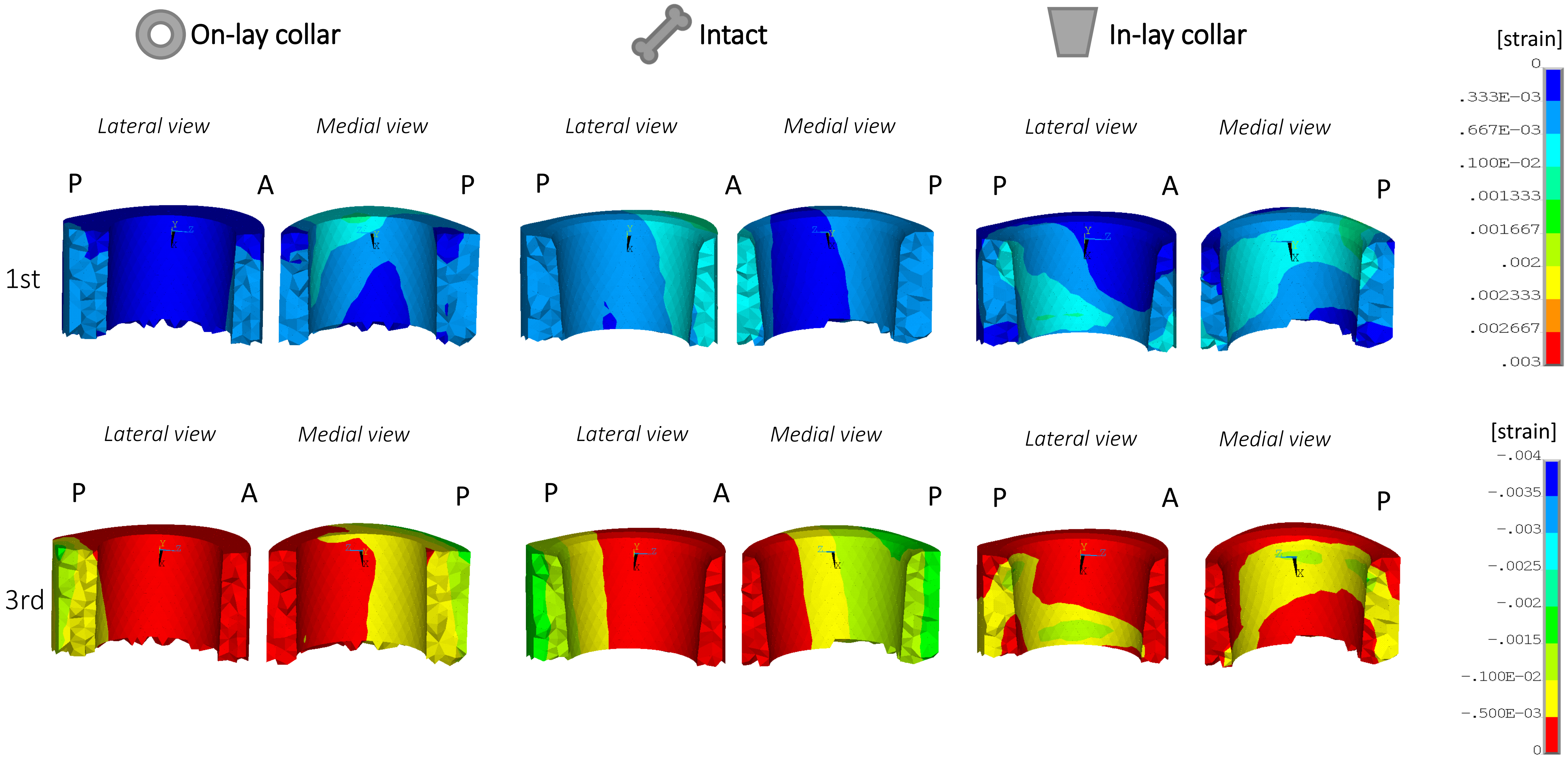

D-PFR / Hoop Strain

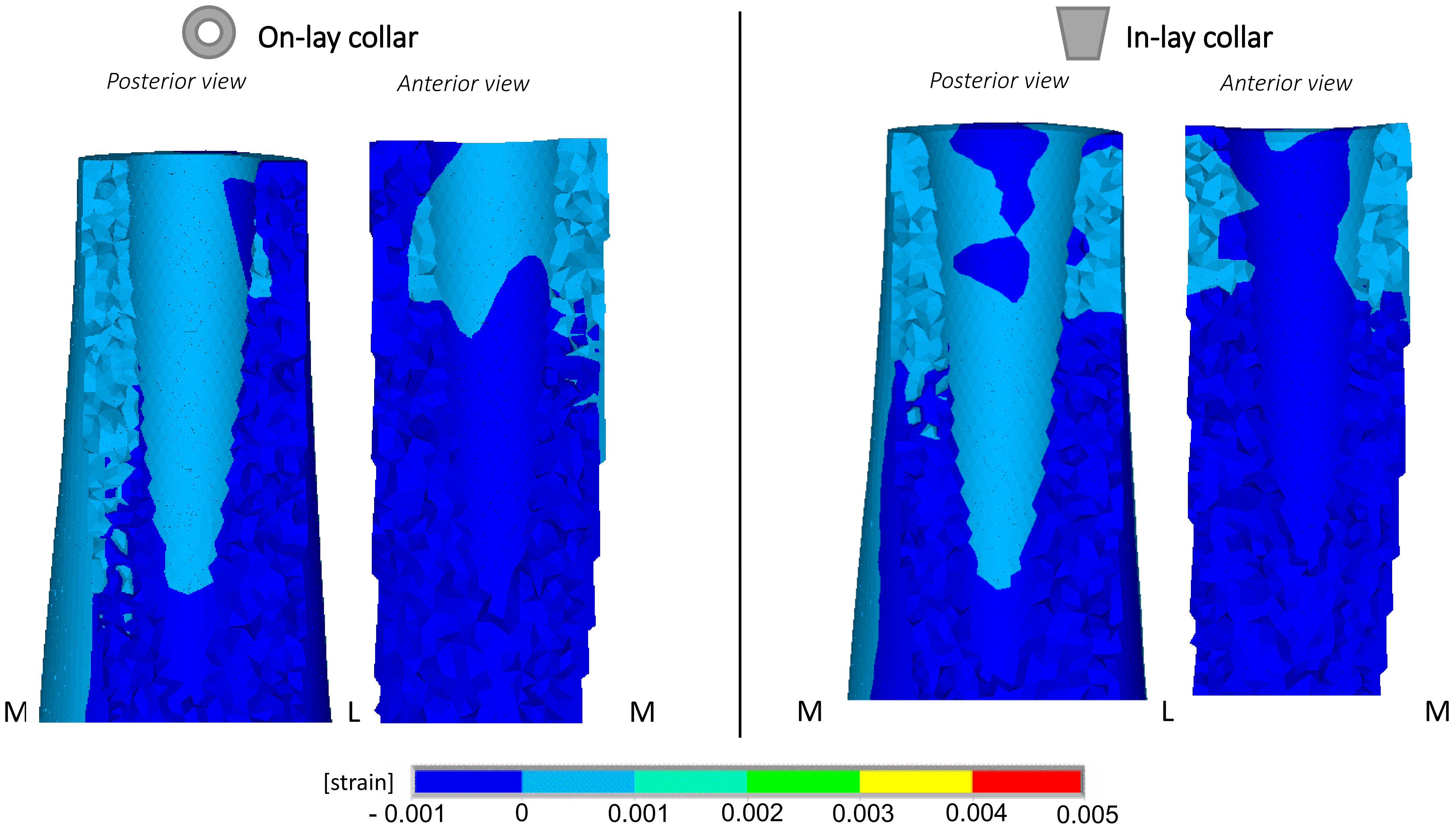

Supplement: Supplementary file 2 [file DataSheet1.PDF]
